# Supplementary material for: Phosphoproteomic Analyses of Interleukin 2 Signaling Reveal Integrated JAK Kinase-Dependent and -Independent Networks in CD8+ T Cells
Source: Immunity. 2016 Sep 20;45(3):685–700. doi: 10.1016/j.immuni.2016.07.022 (PMC5040828; doi:10.1016/j.immuni.2016.07.022)
Supplement: Document S1. Figures S1–S3 and Supplemental Experimental Procedures [file mmc1.pdf]

Immunity, Volume *45*

Supplemental Information

Phosphoproteomic Analyses of Interleukin 2 Signaling Reveal Integrated JAK Kinase-Dependent and -Independent Networks in $CD8^+$ T Cells

Sarah H. Ross, Christina Rollings, Karen E. Anderson, Phillip T. Hawkins, Len R. Stephens, and Doreen A. Cantrell

Figure S1

A

	p-Peptides
Expt 1	6327
Expt 2	5284
Expt 1-2 (shared)	3461 (42.3%)

	p-Peptides
Expt 1	6327
Expt 3	5450
Expt 1-3 (shared)	3482 (41.9%)

	p-Peptides
Expt 2	5284
Expt 3	5450
Expt 2-3 (shared)	4306 (66.9%)

	Expt 1	Expt 2	Expt 3
up regulated	232 (3.66%)	228 (4.31%)	281 (5.15%)
down regulated	306 (4.83%)	262 (4.95%)	201 (3.68%)

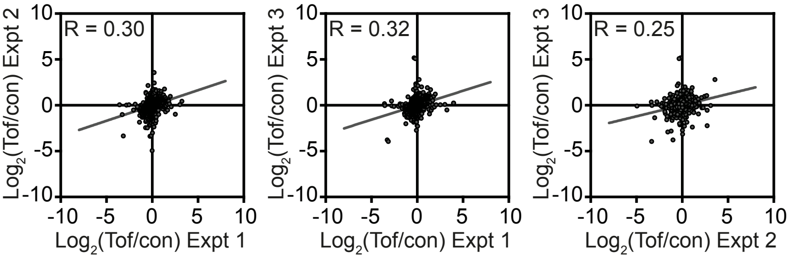

B

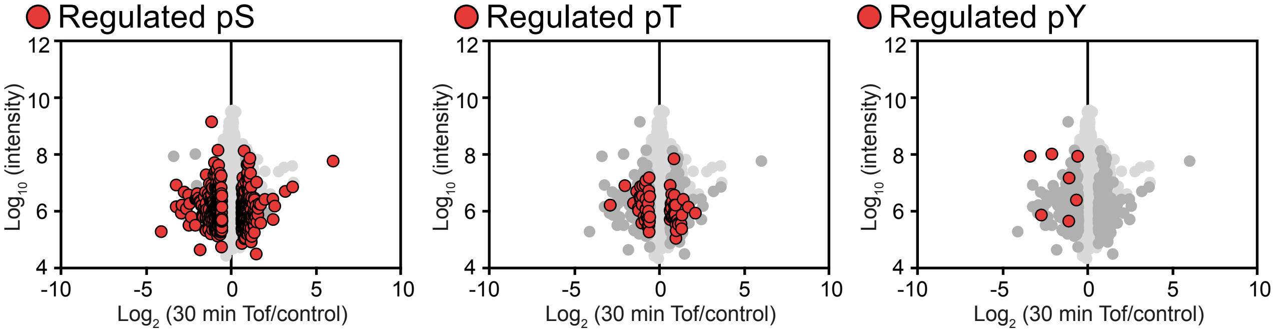

C

	p-Peptides
Expt 1	9934
Expt 2	8733
Expt 1-2 (shared)	7319 (64.5%)

	p-Peptides
Expt 1	9934
Expt 3	5941
Expt 1-3 (shared)	5101 (47.3%)

	p-Peptides
Expt 2	8733
Expt 3	5941
Expt 2-3 (shared)	5040 (52.3%)

	Expt 1	Expt 2	Expt 3
up regulated	247 (2.48%)	176 (2.01%)	98 (1.64%)
down regulated	632 (6.36%)	342 (3.91%)	258 (4.34%)

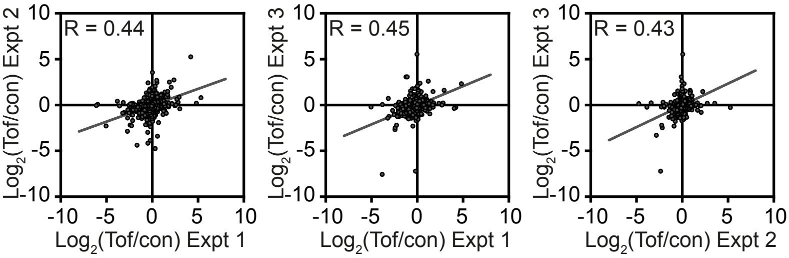

D

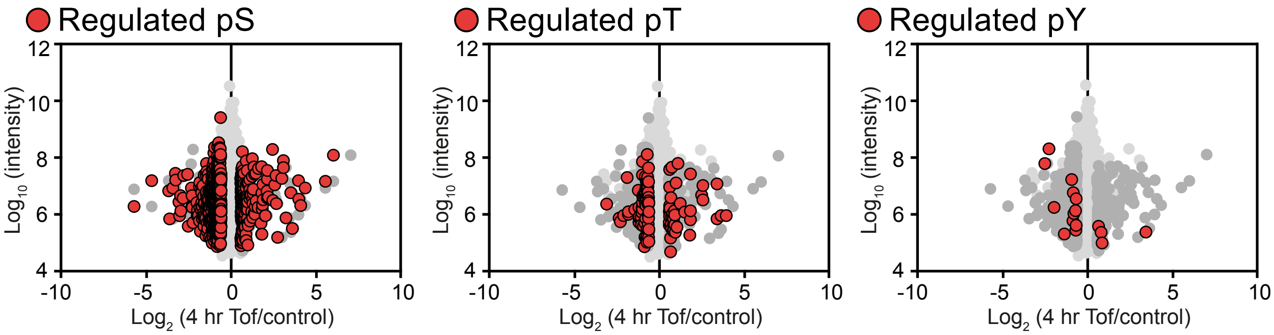

Figure S2

A Selected Lck-Fyn linked pY identified in IL-12 dataset

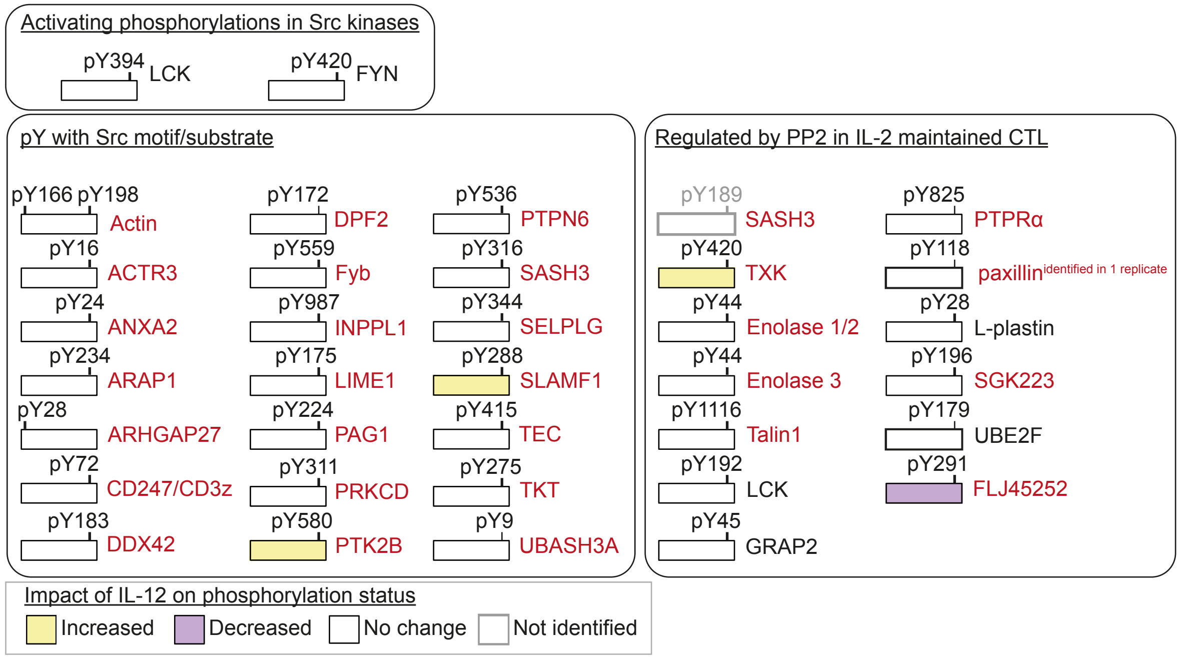

B

	p-Peptides
Expt 1	11937
Expt 2	10796
Expt 1-2 (shared)	8538 (60.1%)

	p-Peptides
Expt 1	11937
Expt 3	12183
Expt 1-3 (shared)	10271 (74.2%)

	p-Peptides
Expt 2	10796
Expt 3	12183
Expt 2-3 (shared)	8621 (60.0%)

	Expt 1	Expt 2	Expt 3
up regulated	521 (4.36%)	392 (3.63%)	615 (5.04%)
down regulated	796 (6.66%)	642 (5.94%)	878 (7.20%)

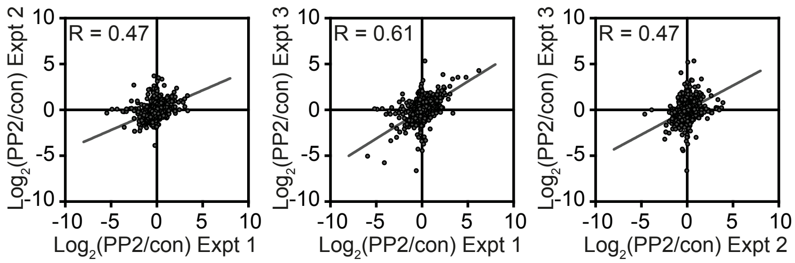

C

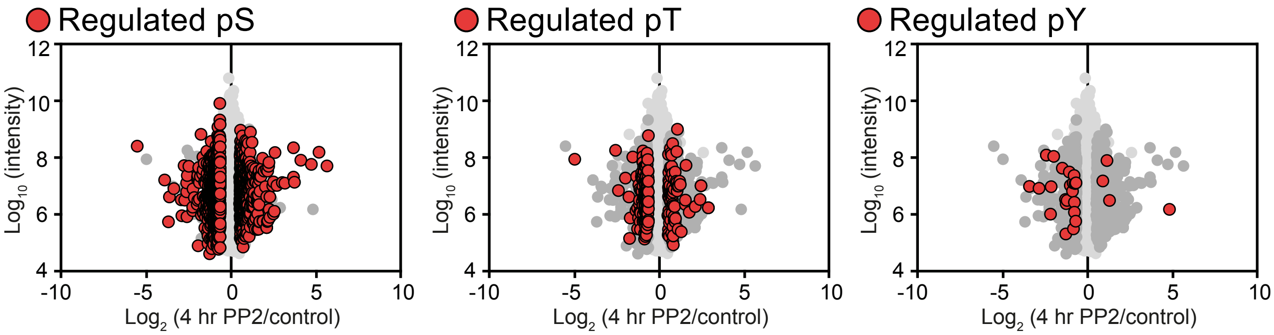

Figure S3

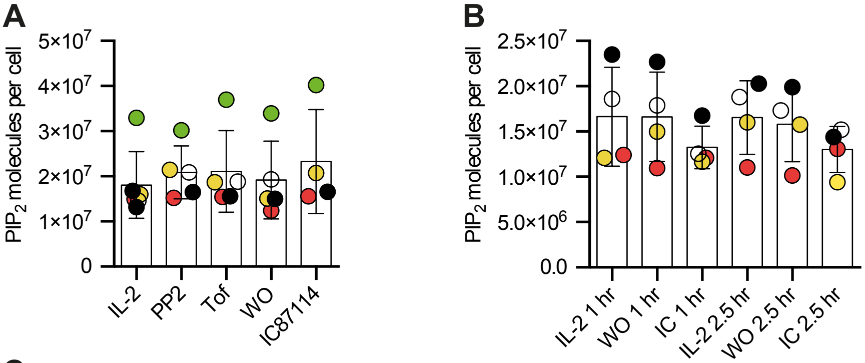

A
PIP2 molecules per cell
5×10^7
4×10^7
3×10^7
2×10^7
1×10^7
0
IL-2
PP2
Tof
WO
IC87114
B
PIP2 molecules per cell
2.5×10^7
2.0×10^7
1.5×10^7
1.0×10^7
5.0×10^6
0.0
IL-2 1 hr
WO 1 hr
IC 1 hr
IL-2 2.5 hr
WO 2.5 hr
IC 2.5 hr

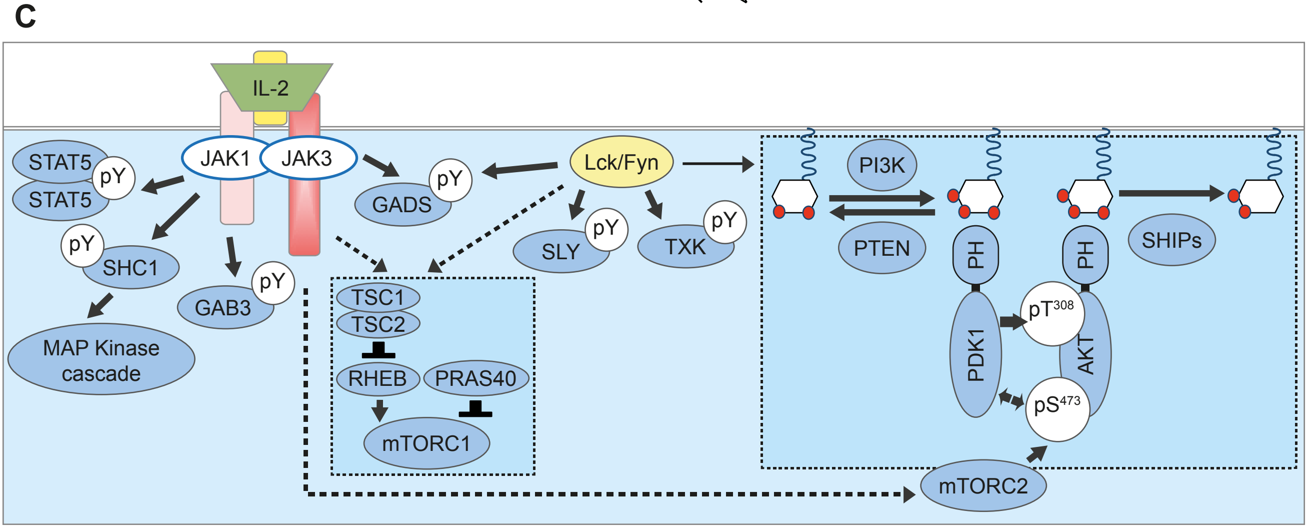

C
IL-2
JAK1
JAK3
STAT5
STAT5
pY
SHC1
MAP Kinase cascade
GAB3
GADS
Lck/Fyn
SLY
TXK
TSC1
TSC2
RHEB
PRAS40
mTORC1
PI3K
PTEN
PH
PDK1
AKT
pT308
pS473
SHIPs
mTORC2

Supplemental Figure Legends

Figure S1. Related to Figure 3: Tofacitinib-regulation of the CTL phosphoproteome.
(A) The overlap and correlation in the SILAC ratios of the phosphosites identified in the individual biological replicates of IL-2 maintained CTL treated with Tofacitinib for 30 minutes. In these experiments, the "heavy"-labeled cells were treated with the inhibitor and the "light" cells were given the control treatment. The numbers and percentages of regulated phosphosites in each replicate are shown alongside. In **(B)**, a graphical representation of all the phosphosites identified in all replicates of the 30 minute Tofacitinib treatment is shown, with log-transformed SILAC ratios plotted against log-transformed peptide intensity. The individual panels show the regulated pS, pT or pY sites in red. In **(C)** the overlap and correlation in the SILAC ratios of the phosphosites identified in the individual biological replicates of IL-2 maintained CTL treated with Tofacitinib for 4 hours is shown. The numbers and percentages of regulated phosphosites in each replicate are shown alongside. The graphs in **(D)** shows the overview of all the phosphosites identified in the three biological replicates of the 4 hour Tofacitinib treatment with log-transformed SILAC ratios plotted against log-transformed peptide intensity, with the red dots in each panel showing the regulated pS, pT or pY sites.

Figure S2. Related to Figure 5: PP2-regulation of the CTL phosphoproteome.
(A) CTL differentiated in "heavy" SILAC media were treated with 20 ng/ml IL-12 for 18 hours in the presence of 20 ng/ml IL-2 and compared to "light" control, CTL maintained in 20 ng/ml IL-2. Cells were processed for mass spectrometry as described in Figure 1A and in Materials and Methods. The data were compared to the IL-2 datasets to extract the SRC kinase regulated/SRC kinase motif-containing phosphorylation sites, and the regulation of those found reproducibly in the 18 hour treatment are shown in **(A)**. **(B)** The overlap and correlation in the SILAC ratios of the phosphosites identified in the individual biological replicates of IL-2 maintained CTL treated with PP2 for 4 hours. The numbers and percentages of regulated phosphosites in each replicate are shown alongside. A graphical representation of all the phosphosites identified in the three biological replicates is shown in **(C)**, with the with log-transformed SILAC ratios plotted against log-transformed peptide intensity. The individual panels show the regulated pS, pT or pY sites in red.

Figure S3. Related to Figure 7: Control of inositol lipid levels in CTL
Mass spectrometry was used to measure the inositol lipid levels in CTL. The average number of molecules of PIP_2 per cell in response to each of the inhibitor treatments was calculated using the mass of the PIP_2 internal standard (ISD), a breakdown product of the PIP_3 ISD. The graph in **(A)** shows the number of PIP_2 per CTL in either IL-2 maintained CTL, or IL-2 maintained CTL treated with 100 nM Tofacitinib (Tof), 10 μM PP2, 10 μM IC87114 or deprived of IL-2 (WO) for 30 minutes. The results for 5 biological replicates are shown. In **(B)** levels of PIP_2 were measured in IL-2 maintained CTL, or those deprived of IL-2 (WO) or treated with IL-2 + IC87114 for up to 2.5 hours. The graph shows the data for 4 biological replicates. In **(A)** and **(B)** individual biological replicate data is shown in matching colors, the bars show the mean ± SD. **(C)** IL-2 dependent-JAK activation stimulates the phosphorylation of a number of signaling scaffold proteins and regulates diverse pT/pS signaling networks in CTL. Cell intrinsic LCK/FYN activity also regulates a number of signaling molecules, notably dominating the regulation of PIP_3 accumulation and the activity of AKT. Signaling integration between JAK and LCK/FYN occurs at multiple levels to regulate signaling that defines T cell function.

Supplemental Table Legends

Table S1. Related to Figure 1: The phosphoproteome of IL-2 stimulated CTL.
All the phosphosites identified in three biological replicates of CTL comparing the phosphoproteome of IL-2 quiesced CTL (con) with IL-2 quiesced CTL treated with 20 ng/ml IL-2 for 15 minutes (IL-2). Phosphosites ratios increased by 1.5-fold or greater are colored yellow and those decreased by 1.5-fold or greater are highlighted in purple. The significance column shows the log-transformed *P* values (one sample t-test). Values ≥ 1.3 are equivalent to *P* value ≤ 0.05.

Table S2. Related to Figure 1 and Figure 2: The IL-2 regulated CTL phosphoproteome.
The phosphosites reproducibly regulated in two or three biological replicates of CTL comparing the phosphoproteome of IL-2 quiesced CTL (con) with quiesced CTL treated with 20 ng/ml IL-2 for 15 minutes (IL-2) are shown. Phosphosites ratios increased by 1.5-fold or greater are colored yellow and

those decreased by 1.5-fold or greater are colored in purple. The significance column shows the log-transformed *P* values (one sample t-test). Values ≥ 1.3 are equivalent to *P* value ≤ 0.05.

Table S3. Related to Figure 1: Functional analysis of IL-2-regulated proteins.
Groupings of proteins whose phosphorylation was reproducibly regulated by IL-2 in two or three biological replicates using DAVID cluster enrichment analysis.

Table S4. Related to Figure 3: The phosphoproteome of IL-2 maintained CTL after 30 minutes of Tofacitinib treatment.
All the phosphosites identified in three biological replicates of CTL comparing the phosphoproteome of IL-2 maintained CTL (con) with IL-2 maintained CTL treated with Tofacitinib (Tof) for 30 minutes. Phosphosites ratios increased by 1.5-fold or greater are colored yellow and those decreased by 1.5-fold or greater are highlighted in purple. The significance column shows the log-transformed *P* values (one sample t-test). Values ≥ 1.3 are equivalent to *P* value ≤ 0.05.

Table S5. Related to Figure 3: The phosphoproteome of IL-2 maintained CTL after 4 hours of Tofacitinib treatment.
All the phosphosites identified in three biological replicates of CTL comparing the phosphoproteome of IL-2 maintained CTL (con) with IL-2 maintained CTL treated with Tofacitinib (Tof) for 4 hours. Phosphosites ratios increased by 1.5-fold or greater are colored yellow and those decreased by 1.5-fold or greater are highlighted in purple. The significance column shows the log-transformed *P* values (one sample t-test). Values ≥ 1.3 are equivalent to *P* value ≤ 0.05.

Table S6. Related to Figure 3: Tofacitinib-regulated phosphosites in CTL.
Lists of phosphosites identified as being regulated by Tofacitinib treatment of IL-2 maintained CTL. Phosphosites were included if they were regulated consistently in both datasets, consistently regulated in the 30 minute dataset but not detected in the 4 hour dataset, consistently regulated following the long-term Tofacitinib treatment but unchanged after 30 minutes of Tofacitinib treatment or if they were consistently regulated after the 4 hour treatment but not identified in the 30 minute treatment. Phosphosites ratios increased by 1.5-fold or greater are colored yellow and those decreased by 1.5-fold or greater are highlighted in purple. The significance columns show the log-transformed *P* values (one sample t-test). Values ≥ 1.3 are equivalent to *P* value ≤ 0.05.

Table S7. Related to Figure 1 and Figure 3: The IL-2 independent phosphoproteome.
The phosphosites identified in the IL-2 stimulated dataset or Tofacitinib datasets but that were not found to be perturbed by the treatments are shown.

Table S8. Related to Figure 4: The CTL tyrosine phosphoproteome.
All phosphotyrosine sites identified in the IL-2 stimulated dataset or Tofacitinib datasets are listed. Those with SRC family kinase motifs, or having been experimentally annotated as being SRC family substrates in the Phosphosite and ELM databases, are highlighted in yellow.

Table S9. Related to Figure 5: The phosphoproteome of IL-2 maintained CTL after 4 hours of PP2 treatment.
All the phosphosites identified in three biological replicates of CTL comparing the phosphoproteome of IL-2 maintained CTL (con) with IL-2 maintained CTL treated with PP2 for 4 hours. Phosphosites ratios increased by 1.5-fold or greater are colored yellow and those decreased by 1.5-fold or greater are highlighted in purple. The significance columns show the log-transformed *P* values (one sample t-test). Values ≥ 1.3 are equivalent to *P* value ≤ 0.05.

Table S10. Related to Figure 5: The PP2 regulated CTL phosphoproteome.
The phosphosites reproducibly regulated in two or three biological replicates of CTL comparing the phosphoproteome of IL-2 maintained CTL (con) with IL-2 maintained CTL treated with PP2 for 4 hours are listed. Phosphosites ratios increased by 1.5-fold or greater are colored yellow and those decreased by 1.5-fold or greater are colored in purple. The significance columns show the log-transformed *P* values (one sample t-test). Values ≥ 1.3 are equivalent to *P* value ≤ 0.05.

Table S11. Related to Figure 6: The integration of IL-2 and LCK/FYN mediated signaling pathways.
The phosphosites reproducibly regulated by both 4 hours of Tofacitinib (Tof) and 4 hours of PP2 treatment. Phosphosites ratios increased by 1.5-fold or greater are colored yellow and those decreased by 1.5-fold or greater are colored in purple.

Supplemental Experimental Procedures

Inhibitors and cell treatments
Tofacitinib (from Selleckchem, Munich, Germany) was used at a concentration of 100 nM. The AKT-1/2 inhibitor (AKTi) (EMD Millipore) was used at 1 μM, the PI3K-p110δ inhibitor IC87114 (made in-house) and PP2 (Tocris) were used at 10 μM. Where appropriate, cells were treated with DMSO as controls. To deprive cells of IL-2, cells were pelleted, washed in pre-warmed media lacking IL-2 for 5 minutes before being pelleted and resuspended in pre-warmed media lacking IL-2. To standardize, the time course of IL-2 deprivation started when the first media wash lacking IL-2 was added to the cells.

Cell counts
Accurate cell counts for mixing "light" and "heavy" labeled cells for the phosphoproteomic studies were obtained using a FACSVerse flow cytometer with FACSuite software (BD Biosciences). Viable cells were gated according to their forward- and side-scatter profiles and counts were determined using the FACSuite software or with FlowJo software (Treestar).

Sample preparation, phosphopeptide enrichment and mass spectrometry
For the IL-2 stimulation of CTL, cells were labeled in "light" or "heavy" media supplemented with 20 ng/ml IL-2 for 4 days. Cells were then starved of IL-2 for 24 hours. To remove IL-2, cells were washed once in pre-warmed Lysine and Arginine free media lacking IL-2, before being resuspended in the appropriated "light" or "heavy" media lacking IL-2. Cells were supplemented with 20 ng/ml IL-12 (R&D Systems) during this time to support cell viability and the expression of the IL-2 receptor alpha chain. After 24 hours, the "heavy" cells were treated with 20 ng/ml IL-2 for 15 minutes. The control, "light" samples were given a mock stimulation.

For the Tofacitinib and PP2 studies, three independent biological replicates of P14 CTLs maintained in 20 ng/ml IL-2 only were analysed. The "light" condition was used for the control cells, and the P14 CTL labeled in "heavy" SILAC media for 5 days were treated with the inhibitor.

Following the treatments, the control and treated cells were mixed at 1:1 ratio (total $2x10^8$ cells). Cells were pelleted and washed once in cold PBS and lysed in 8 M urea, 50 mM Tris-HCl pH 8 and 1 mM TCEP (Pierce). The lysed samples were then sonicated and the proteins were precipitated with trichloroacetic acid (10%, v/v) for 15 minutes at room temperature. The resulting protein pellets were washed thoroughly before being resuspended in 8M urea, 50 mM Tris-HCl pH 8. The proteins were then subjected to alkylation with iodoacetamide (Sigma-Aldrich) prior to digestion with trypsin (Promega). Proteolytic digestion products were desalted using C18 Sep-Pak cartridges (Waters), and fractionated by hydrophilic interaction liquid chromatography (HILIC).

Hydrophilic interaction liquid chromatography (HILIC) and phosphopeptide enrichment.
Desalted tryptic peptides were fractionated using Ultimate 3000 HPLC (Thermo Scientific) equipped with a 4.6 × 250- mm TSKgel Amide-80 5-μm particle column (Tosoh Biosciences). For the separation, the buffers used were 0.1% TFA (HILIC buffer A) and 99.9% acetonitrile, 0.1% TFA (HILIC buffer B). The peptide samples were resuspended in 80% HILIC buffer B and injected onto the HILIC column. The chromatography was performed using the following elution gradient: 80% B held for 20 minutes followed by 80% B to 60% B in 40 minutes and finally 0% B for 10 minutes at a flow rate of 0.4 ml/minute. In total, 16 phosphopeptide fractions were collected.

Phosphopeptides were enriched using previously described protocols. For the Tofacitinib and PP2 treatments, titanium dioxide (Titansphere, GL Science) (Larsen et al., 2005; Thingholm et al., 2006) was used for the enrichment and for IL-2 dataset Ti-IMAC (MagReSyn) (Tape et al., 2014) was used. In each case, phosphopeptides were eluted with 200 μl 0.4 M NH_4OH followed with 200 μl 0.2 M NH_4OH/50% acetonitrile and then dried using a speedvac (Genevac).

Liquid Chromatography-Mass Spectrometry

Phosphopeptide samples were resuspended in 1% formic acid and separated by nanoscale C18 reverse-phase liquid chromatography (Ultimate 3000 RSLC nano system, Thermo Scientific). The buffers used for chromatography were: HPLC Buffer A (2% acetonitrile, 0.1% formic acid), HPLC Buffer B (80% acetonitrile, 0.08% formic acid) and HPLC Buffer C (0.1% formic acid). Samples were washed onto the column with HPLC Buffer C and eluted with the following buffer gradient: 2% B (0-3 minutes), 2-40% B (3-128 minutes), 40-98% B (128-130 minutes), 98% B (130-150 minutes), 98-2% B (150-151 minutes), and equilibrated in 2% B (151-180 minutes) at a flow rate of 0.3 μl/minute. The eluting peptide solution was automatically electrosprayed into the coupled Linear Trap Quadrupole (LTQ)-Orbitrap mass spectrometer (LTQ-Orbitrap Velos Pro; Thermo Scientific) using an Easy-Spray nanoelectrospray ion source (Thermo Scientific). The mass spectrometers were operated in positive ion mode and were used in data-dependent acquisition modes. A full scan (FT-MS) was acquired at a target value of 1,000,000 ions with resolution R = 60,000 over a mass range of 335-1800 atomic mass unit (amu). The fifteen most intense ions were selected for fragmentation in the LTQ Orbitrap Velos. Fragmentation in the LTQ was induced by collision-induced dissociation (CID) with a target value of 10,000 ions. For accurate mass measurement, the "lock mass" function (lock mass = 445.120024 Da) was enabled for MS scan modes. To improve the fragmentation of phosphopeptides, the multistage activation algorithm in the Xcalibur software was enabled for each MS/MS spectrum using the neutral loss values of 97.98, 48.99, 32.66 and 24.49 m/z units. Former target ions selected for MS/MS were dynamically excluded for 45 seconds. General mass spectrometric conditions were as follows: spray voltage, 1.8-2.5 kV; no sheath and auxiliary gas flow; ion transfer tube temperature, 250 °C; normalised collision energy (35%) using wide band activation mode for MS2. The isolation width was set to 2 amu for IT-MS/MS. Ion selection thresholds were 5000 counts for MS2. An activation of q = 0.25 and activation time of 10 ms were applied in MS2 acquisitions. The fill time for FTMS was set to 500 ms and for ITMS to 100 ms.

Data processing and Bioinformatics

Mass spectrometry data were processed in MaxQuant version 1.5.0 using the following search parameters: a MS tolerance of 20 ppm, MS/MS tolerance of 0.5 Da and full trypsin specificity. Up to two missed cleavages were permitted. Protein N-terminal acetylation, oxidation of methionine, glutamine to pyroglutamate conversion, deamidation (NQ) and phosphorylation of serine, threonine and tyrosine were set as variable modifications, while carbamidomethylation of cysteine was set as fixed modification. The minimum peptide length for identification was set to at least 6 amino acids in length. The match between run function was enabled. False discovery rates (FDRs) of 0.01 were based on hits against a reversed sequence database and calculated at the level of peptides, proteins and modification sites.

Perseus software was used to map identified phosphorylation sites with kinase motifs, and known kinase-substrate interactions obtained from the mouse and human PhosphoSitePlus (Hornbeck et al., 2015) database and the human phosphoELM (Dinkel et al., 2016) database. In addition, Perseus software was used to annotate the identified phosphoproteins with Gene Ontology (GO) biological processes (BP) and molecular functions (MF).

Phosphosites were considered to be up regulated if the SILAC ratio was greater than 1.5 and down regulated if the SILAC ratio was less than 0.667. Reproducibly regulated phosphorylation sites had to be identified in at least two of the three biological replicates and regulated consistently where identified. Statistical analysis of the Log_2 transformed ratio changes was performed using a one-sample t-test in Perseus. Regulated phosphorylation sites were not selected based on a statistical cut-off to ensure that all potentially regulated sites would be considered when comparing datasets. When phosphosites were identified in multiple biological replicates, the mean SILAC ratio of the replicates was used to present data.

Phosphosites considered to be regulated by Tofacitinib were either regulated consistently in both the 30 minute and 4 hour treatment datasets; consistently regulated in the 30 minute dataset but not detected in the 4 hour dataset; consistently regulated following the long-term Tofacitinib treatment but unchanged after 30 minutes of Tofacitinib treatment; consistently regulated after the 4 hour treatment but not identified in the 30 minute treatment.

When comparing the impact of different treatments on the CTL phosphoproteome, only phosphorylation sites reproducibly identified in both datasets were compared.

MaxQuant reports phosphosite positions for all potential isoforms of the relevant protein: in figures, where possible, the numbering for the first protein isoform is shown rather than the first phosphosite reported in the data table.

The proteins identified as being regulated by each treatment were subjected to functional analysis using DAVID bioinformatics resources to search for associated GO-BP and GO-MF terms. For enrichment analysis using DAVID, the proteins regulated by each treatment were compared to a custom background comprised of all phosphoproteins identified in the dataset (regulated and non-regulated). The combined BP and MF GO annotations were then grouped using the functional annotation clustering tool, with a medium clustering stringency and an EASE (Expression analysis systematic explorer) score, a modified Fisher Exact *P*-value, of ≤0.1.

Western blotting
Cells were lysed in ice cold lysis buffer (50 mM HEPES, pH 7.4, 150 mM sodium chloride, 1% (w/v) NP-40, 0.5% (w/v) sodium deoxycholate, 0.1% (w/v) SDS, 10% (w/v) glycerol, 1 mM EDTA, 50 mM sodium fluoride, 5 mM sodium pyrophosphate, 10 mM sodium β-glycerophosphate, 0.5 mM sodium orthovanadate, 5 mM N-ethylmaleimide, 1 mM TCEP (Pierce), with protease and phosphatase inhibitor tablets (Roche) at a concentration of 20 million CTL per ml lysis buffer. Lysates were sonicated on ice and centrifuged (16,000.g for 10 minutes at 4°C) before being mixed with NuPAGE LDS sample buffer (Life Technologies) supplemented with TCEP and separated by gel electrophoresis (Bio-Rad Mini-PROTEAN tetra cell system). Proteins were then transferred to nitrocellulose membrane (Protran), using standard conditions (Hukelmann et al., 2016). Membranes were blocked with 5% (w/v) Bovine Serum albumen and 1% (w/v) non-fat dried skimmed milk powder in phosphate-buffered saline (PBS) containing 0.05% Tween 20.

Membranes were probed with the following primary antibodies: STAT5A/B (CST #9363), STAT5A/B pY694/699 (CST #9351), S6 pS235/236 (CST #2211), YB-1 (YBX1) pS102 (CST #2900), AKT (CST #9272), AKT pT308 (CST #4056), AKT pS473 (CST #4058), SMC1 (Bethyl Laboratories, A300-055A), FOXO1/3A pT24/32 (CST #9464), FOXO1 (CST #9454), p44/42 (Erk1/2) (clone 3A7, CST #9107), p44/42 (ERK1/2) pT202/pY204 (clone E10, CST #9106). Primary antibodies were detected using HRP-conjugated secondary antibodies (goat anti rabbit, Thermo Scientific #31460; goat anti mouse, Thermo Scientific #31430), and chemiluminescence was measured using X-ray films (Konica) or an Odyssey Fc Imaging System (Licor).

Cell staining and flow cytometry
For surface markers, cells were washed once in 0.5% FBS (v/v) in PBS and stained for 20 minutes at 4°C in the same solution with saturating concentrations of antibodies as described previously (Navarro et al., 2012). Antibodies used were CD8α (clone 53-6.7) coupled to Horizon V450 (HV450) and CD25 (clone PC61) coupled to phycoerythrin-cyanine 7 (PE-Cy7) from BD Biosciences.

For intracellular staining of phospho-S6, cells were treated with or without inhibitors as appropriate, with control cells being treated 20 nM rapamycin for 30 minutes. Following treatments, cells were fixed in 1% (w/v) paraformaldehyde at 37ºC for 15 minutes. Cells were then washed in 0.5% FBS (v/v) in PBS removed from the fixation buffer and permeabilised and incubated with 90% (v/v) methanol at -20ºC for at least 30 minutes. Following permeabilisation, cells were washed twice and incubated with antibody against S6 pS235/pS236 (CST #2211) for 30 minutes at room temperature. Following staining, cells were washed twice and incubated with Alexa 647-conjugated anti-rabbit secondary antibody (CST #4414) for 30 minutes at room temperature. Cells were washed twice and resuspended in 0.5% FBS (v/v) in PBS for acquisition.

For O-propargyl-puromycin (OPP, Jena Bioscience) incorporation analysis, cells were stimulated as appropriate, with control cells being pre-treated with cycloheximide for 30 minutes. Following labeling with 20 μM OPP for 10 minutes, cells were fixed with 1% paraformaldehyde for 15 minutes at room temperature. Cells were washed in 0.5% FBS (v/v) in PBS before being permeabilised with 0.5% Triton X-100 (v/v) in PBS for 15 minutes at room temperature. The incorporated OPP was then labeled with Alexa 647-azide (Invitrogen) using a standard Click-IT chemistry reaction (Invitrogen). Cells were then washed twice with 0.5% FBS (v/v) in PBS, before being analysed.

Data were acquired on a FACS LSR Fortessa flow cytometer with DIVA software (BD Biosciences) or FACSVerse flow cytometer with FACSuite software (BD Biosciences). Viable cells were gated according to their forward- and side-scatter profiles. Data analysis was performed with FlowJo software (Treestar).

Mass spectrometry measurements of inositol lipids

The analysis of inositol lipids was performed as described previously (Clark et al., 2011), using a QTRAP 4000 (AB Sciex) mass spectrometer and employing the lipid extraction and derivatization method described for cultured cells, with the modification that 10 ng C17:0/C16:0 PIP_3 internal standard (ISD) and 100 ng C17:0/C16:0 phosphatidylinositol (PI) ISD were added to primary extracts, and that final samples were dried in a speedvac concentrator rather than under N_2. PIP_3 responses were normalised to PIP_2 responses. The mass of the PIP_2 ISD, a breakdown product of the PIP_3 ISD, was calculated using the PIP_3 internal standard (ISD), and then was used to calculated the average number of PIP_2 molecules per cell in response to each of the inhibitor treatments.

Confocal microscopy

Cells were allowed to attach to coverslips coated with poly-l-lysine (Sigma-Aldrich), then fixed for 30 minutes at 25°C with 4% (w/v) paraformaldehyde. Following fixation, cells were permeabilised using 0.1% Triton X-100 for 5 minutes. Actin was visualized using phalloidin coupled to Alexa fluor 647 (Invitrogen). A Zeiss LSM700 confocal microscope with an alpha Plan-FLUAR 100× objective (numerical aperture, 1.45) was used for imaging.

Supplemental References

Clark, J., Anderson, K.E., Juvin, V., Smith, T.S., Karpe, F., Wakelam, M.J.O., Stephens, L.R., and Hawkins, P.T. (2011). Quantification of PtdInsP3 molecular species in cells and tissues by mass spectrometry. Nat. Methods *8*, 267–272.

Dinkel, H., Van Roey, K., Michael, S., Kumar, M., Uyar, B., Altenberg, B., Milchevskaya, V., Schneider, M., Kühn, H., Behrendt, A., et al. (2016). ELM 2016--data update and new functionality of the eukaryotic linear motif resource. Nucleic Acids Res. *44*, D294–D300.

Hornbeck, P.V., Zhang, B., Murray, B., Kornhauser, J.M., Latham, V., and Skrzypek, E. (2015). PhosphoSitePlus, 2014: mutations, PTMs and recalibrations. Nucleic Acids Res. *43*, D512–D520.

Hukelmann, J.L., Anderson, K.E., Sinclair, L.V., Grzes, K.M., Murillo, A.B., Hawkins, P.T., Stephens, L.R., Lamond, A.I., and Cantrell, D.A. (2016). The cytotoxic T cell proteome and its shaping by the kinase mTOR. Nat. Immunol. *17*, 104–112.

Larsen, M.R., Thingholm, T.E., Jensen, O.N., Roepstorff, P., and Jørgensen, T.J.D. (2005). Highly selective enrichment of phosphorylated peptides from peptide mixtures using titanium dioxide microcolumns. Mol. Cell Proteomics *4*, 873–886.

Navarro, M.N., Sinclair, L.V., Feijoo-Carnero, C., Clarke, R., Matthews, S.A., and Cantrell, D.A. (2012). Protein kinase D2 has a restricted but critical role in T-cell antigen receptor signalling in mature T-cells. Biochem. J. *442*, 649–659.

Tape, C.J., Worboys, J.D., Sinclair, J., Gourlay, R., Vogt, J., McMahon, K.M., Trost, M., Lauffenburger, D.A., Lamont, D.J., and Jørgensen, C. (2014). Reproducible automated phosphopeptide enrichment using magnetic TiO2 and Ti-IMAC. Anal Chem *86*, 10296–10302.

Thingholm, T.E., Jørgensen, T.J.D., Jensen, O.N., and Larsen, M.R. (2006). Highly selective enrichment of phosphorylated peptides using titanium dioxide. Nat Protoc *1*, 1929–1935.
