# Supplementary material for: Phosphoproteomic Analyses of Interleukin 2 Signaling Reveal Integrated JAK Kinase-Dependent and -Independent Networks in CD8+ T Cells
Source: Immunity. 2016 Sep 20;45(3):685–700. doi: 10.1016/j.immuni.2016.07.022 (PMC5040828; doi:10.1016/j.immuni.2016.07.022)
Supplement: Document S2. Article plus Supplemental Information [file mmc13.pdf]

Resource

Immunity

Phosphoproteomic Analyses of Interleukin 2 Signaling Reveal Integrated JAK Kinase-Dependent and -Independent Networks in CD8$^+$ T Cells

Graphical Abstract

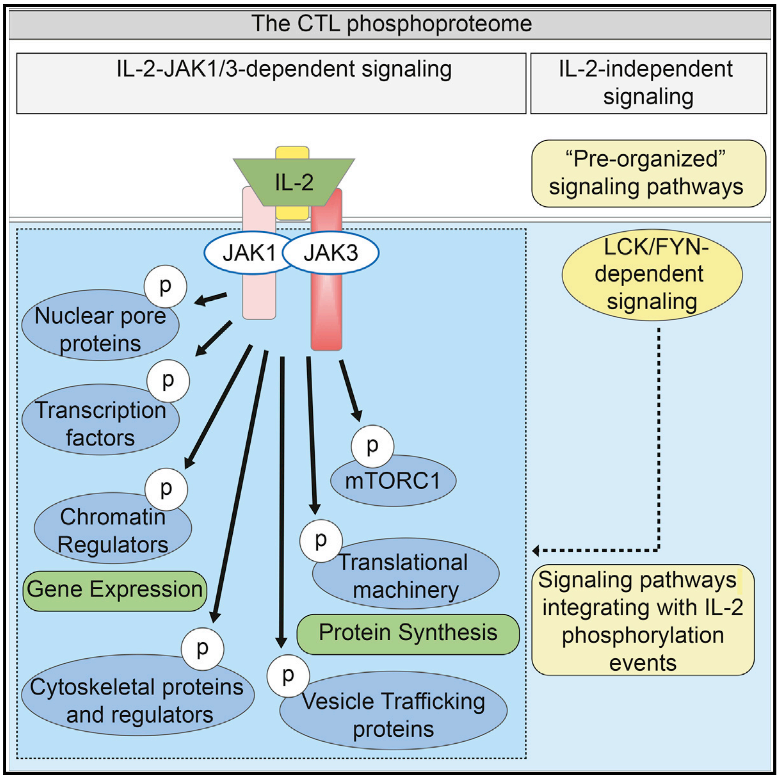

Authors

Sarah H. Ross, Christina Rollings, Karen E. Anderson, Phillip T. Hawkins, Len R. Stephens, Doreen A. Cantrell

Correspondence

d.a.cantrell@dundee.ac.uk

In Brief

Ross et al. define the interleukin-2 (IL-2)-regulated phosphoproteome of cytotoxic T lymphocytes (CTLs) and reveal that the IL-2-JAK1/3 axis integrates with "pre-organized" phosphorylation networks to drive signaling that determines CTL fate. These data present a resource for further examination of IL-2 signaling pathways.

Highlights

- Analysis of IL-2-JAK phosphorylation networks in cytotoxic T lymphocytes (CTL)
- Over 900 phosphorylations on more than 600 proteins regulated in response to IL-2
- SRC family kinases signal in CTLs independently of IL-2 in "pre-organized" networks
- Both IL-2-JAK and IL-2-independent signaling regulates pathways that define CTL fate

Accession Numbers

PXD004645
PXD004644

Ross et al., 2016, Immunity 45, 685–700
September 20, 2016 © 2016 The Author(s). Published by Elsevier Inc.
http://dx.doi.org/10.1016/j.immuni.2016.07.022

Phosphoproteomic Analyses of Interleukin 2 Signaling Reveal Integrated JAK Kinase-Dependent and -Independent Networks in CD8$^+$ T Cells

Sarah H. Ross,[1] Christina Rollings,[1] Karen E. Anderson,[2] Phillip T. Hawkins,[2] Len R. Stephens,[2] and Doreen A. Cantrell[1,3,*]
[1]Division of Cell Signalling and Immunology, School of Life Sciences, University of Dundee, Dundee DD1 5EH, UK
[2]Inositide Laboratory, Babraham Institute, Babraham Research Campus, Cambridge CB22 3AT, UK
[3]Lead Contact
*Correspondence: d.a.cantrell@dundee.ac.uk
http://dx.doi.org/10.1016/j.immuni.2016.07.022

SUMMARY

Interleukin-2 (IL-2) is a fundamental cytokine that controls proliferation and differentiation of T cells. Here, we used high-resolution mass spectrometry to generate a comprehensive and detailed map of IL-2 protein phosphorylations in cytotoxic T cells (CTL). The data revealed that Janus kinases (JAKs) couple IL-2 receptors to the coordinated phosphorylation of transcription factors, regulators of chromatin, mRNA translation, GTPases, vesicle trafficking, and the actin and microtubule cytoskeleton. We identified an IL-2-JAK-independent SRC family Tyr-kinase-controlled signaling network that regulates ~10% of the CTL phosphoproteome, the production of phosphatidylinositol (3,4,5)-trisphosphate (PIP_3), and the activity of the serine/threonine kinase AKT. These data reveal a signaling framework wherein IL-2-JAK-controlled pathways coordinate with IL-2-independent networks of kinase activity and provide a resource toward the further understanding of the networks of protein phosphorylation that program CTL fate.

INTRODUCTION

Interleukin-2 (IL-2) directs the clonal expansion and differentiation of CD4$^+$ and CD8$^+$ regulatory, effector, and memory T cell populations. The IL-2 receptor (IL-2R) comprises IL-2Rβ:IL-2Rγ heterodimers and CD25, the α chain, which confers high-affinity binding of IL-2 to the receptor (Liao et al., 2013; O'Shea et al., 2015). By coupling to the Janus family kinases JAK1 and JAK3, the IL-2R controls the Tyr phosphorylation and activation of Signal Transducer and Activator of Transcription 5 (STAT5) transcription factors (Liao et al., 2013; O'Shea et al., 2015). The biological importance of IL-2 has prompted interest in therapeutic manipulation of IL-2 signaling. Drugs to block IL-2-JAK signaling are one approach to modulate adaptive immune responses (O'Shea et al., 2015; Smith et al., 2016), but there is increasing interest in using engineered cytokines or cytokine antibodies to selectively modulate, rather than ablate, IL-2 signaling (Arenas-Ramirez et al., 2015; Mitra et al., 2015; Spangler et al., 2015).

Rational manipulation of IL-2 signaling is limited by the lack of information about its full complexity. In particular, there is growing recognition that IL-2 signaling extends beyond STATs and includes signaling networks controlled by guanosine-5′-triphosphate hydrolases (GTPases) and lipid and serine/threonine (Ser/Thr) kinases. IL-2-induced metabolic and transcriptional programs are regulated by the Ser/Thr kinase, mammalian target of rapamycin complex 1 (mTORC1) (Finlay et al., 2012; Ray et al., 2015). IL-2 also drives the accumulation of active, guanosine-5′-triphosphate (GTP)-bound Ras GTPases and activation of the Raf-MAPK-ERK MAP kinase cascade (Liao et al., 2013). Moreover, IL-2-maintained T cells accumulate phosphatidylinositol (3,4,5)-trisphosphate (PIP_3) (Cornish et al., 2006), the product of phosphatidylinositol 3-kinases (PI3K). This lipid binds to the pleckstrin homology (PH) domain of the Ser/Thr kinase AKT and coordinates its activation by phosphoinositide-dependent protein kinase-1 (PDK1) (Najafov et al., 2012). The strength and duration of AKT activity then direct T cell transcriptional programs that determine T cell fate (Macintyre et al., 2011).

In terms of the potential complexity of IL-2 signaling, IL-2-maintained cytotoxic T cells (CTLs) express ~250 protein kinases and 120 protein phosphatases (Hukelmann et al., 2016). However, the details of how IL-2R occupancy impacts protein phosphorylation networks in CTLs are not mapped. Moreover, while JAK1 and JAK3 are critical for IL-2 signal transduction, it is not known if the IL-2R couples to Ser/Thr kinases solely by JAK activation. Thus, IL-2 signaling has been reported to involve SRC family kinases such as lymphocyte-specific protein tyrosine kinase (LCK) (Hatakeyama et al., 1991; Horak et al., 1991; Kobayashi et al., 1993) and FYN (Kobayashi et al., 1993). The importance of intrinsic SRC family kinase activity for antigen receptor signaling in T cells is well documented (Chang et al., 2016; Nika et al., 2010). In contrast, the role of SRC kinases in cytokine signaling processes is not understood.

Accordingly, we used mass-spectrometry-based quantitative phosphoproteomics to explore IL-2-controlled protein phosphorylation pathways in primary CD8$^+$ effector cytotoxic T cells where IL-2 directs the transcriptional programs that control proliferation and effector functions. Our data mapped many previously unrecognized IL-2-controlled phosphorylations and uncovered the diversity and intricacy of signaling pathways influenced by IL-2. We also detected a network of IL-2-independent

This is an open access article under the CC BY license (http://creativecommons.org/licenses/by/4.0/).

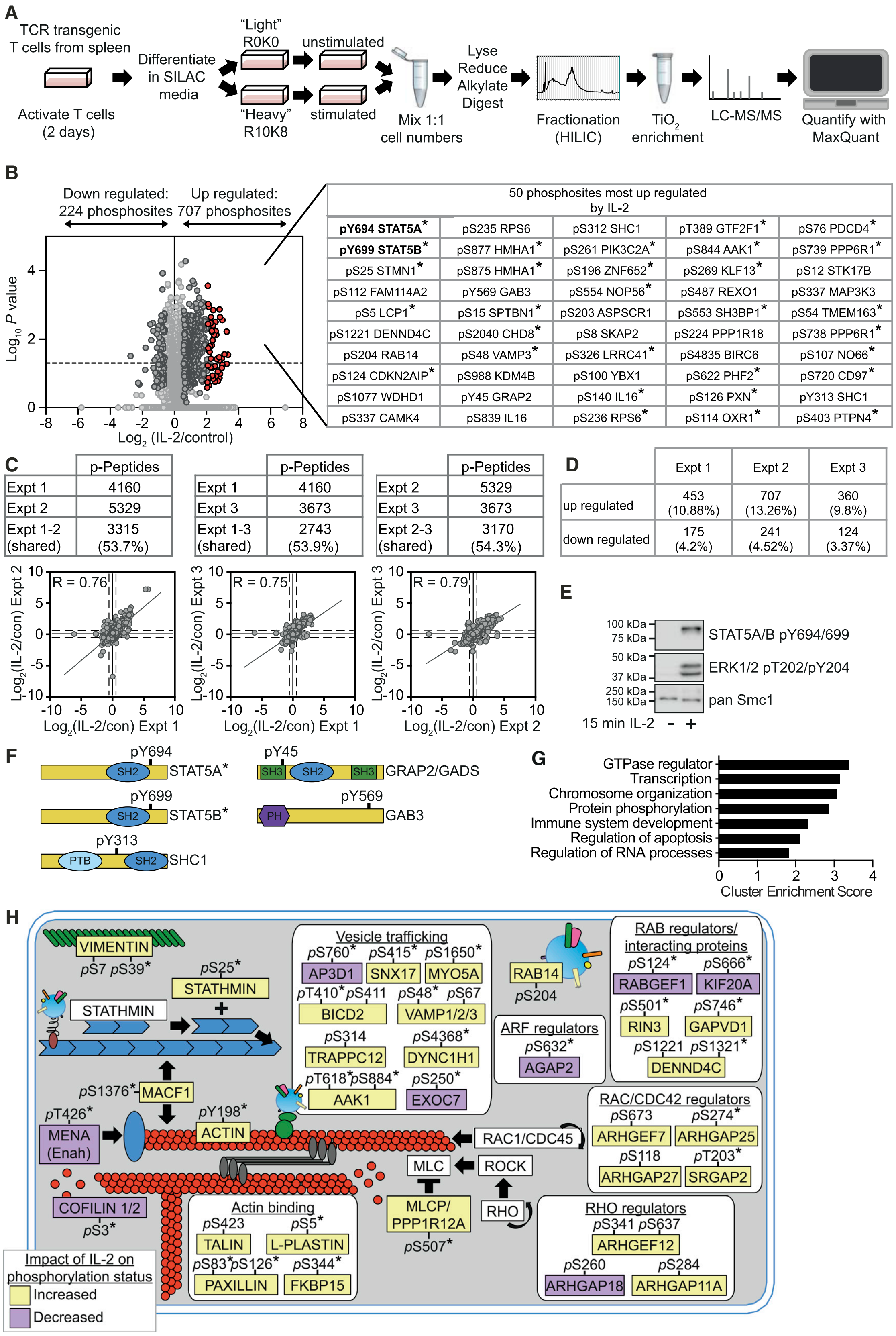

(legend on next page)

phosphorylations mediated by SRC family kinases in CTLs. This JAK-independent signaling controlled PIP_3 levels and AKT activity in CTLs. Our data provide a valuable resource of IL-2-mediated phosphorylations and force a revision of the models of IL-2 as a signaling switch for PIP_3-AKT signaling. Additionally, the data give new ideas for therapeutic modulation of key pathways that control CTL fate.

RESULTS

The IL-2-Regulated Phosphoproteome

To produce effector CTLs, antigen-primed P14 TCR transgenic $CD8^+$ T cells were clonally expanded in IL-2. For phosphoproteomic work, we used SILAC and quantitative high-resolution mass spectrometry following a protocol where CTLs were lysed and digested with trypsin and phosphopeptides enriched by HILIC fractionation followed by TiO_2 affinity chromatography and analysis on an LTQ-Orbitrap Velos (Figure 1A). To examine IL-2-regulated phosphorylations, CTLs were quiesced by 24 hr of IL-2 deprivation and then rechallenged with IL-2 for 15 min. One complication of IL-2 deprivation experiments in primary non-transformed T cells is that IL-2 is required for CTL survival and for the expression of the high-affinity IL-2R complex. Accordingly, we cultured IL-2-deprived CTLs in IL-12, which maintains cell viability and supports expression of IL-2Rs and IL-2 responsiveness. The collective analysis of data from three biological replicates of IL-2-deprived versus IL-2-stimulated CTLs identified 6,458 phosphorylations on 2,248 proteins (Figure 1B and Table S1). The total number of phosphosites identified in the individual biological replicates was similar (Figure 1C). In each replicate, IL-2 increased phosphorylation on ~10% of the identified phosphorylation sites and decreased ~4% of the quantified phosphorylations (Figure 1D). Activation of IL-2 signaling was verified in all three replicates by the reproducible detection of a robust increase in phosphorylation of STAT5A pY694 and STAT5B pY699. Western blot analysis also confirmed the IL-2 responsiveness of IL-2-deprived and IL-12-maintained CTLs, showing strong induction of STAT5A pY694, STAT5B pY699, and ERK1 and/or ERK2, pT202, and pY204 phosphorylations (Figure 1E). Collectively, these experiments identified ~700 phosphosites that were increased and ~220 phosphosites that were decreased in response to IL-2 stimulation of CTLs (Figure 1B). Thus, IL-2 both positively and negatively regulated protein phosphorylations in CTL.

This phosphoproteome analysis revealed that evaluating STAT5A pY694 and STAT5B pY699 phosphorylation gives a limited perspective of IL-2 signaling. For example, IL-2 induced a strong Tyr phosphorylation of three adaptor proteins: GRAP2/GADS (pY45), GAB3 (pY569), and SHC1 (pY313) (Figure 1F). However, our dataset provided an extensive mapping of the impact of IL-2 on the Ser/Thr phosphoproteome. For example, phosphorylation of Ser/Thr kinases, STK17B (DRAK2) and CAMKIV, was reproducibly increased by IL-2. The Ser phosphorylation of Stathmin, a protein that controls microtubule assembly, and the actin regulator, L-plastin (LCP1), was increased with a similar magnitude to STAT5 Tyr phosphorylation (Figure 1B and Table S2). The functional diversity of IL-2-regulated phosphoproteins was striking; IL-2 equally targeted proteins linked to gene transcription and regulators of GTPases and RNA (Figure 1G and Table S3). Notably, the regulation of GTPase-activating proteins (GAPs) and guanine nucleotide exchange factors (GEFs) for Rac, CDC42, and RHO, as well as Cofilin1 and 2, and actin (Figure 1H and Table S3) revealed that IL-2 signaled to the actin cytoskeleton. IL-2-controlled phosphorylation of regulators of ADP ribosylation factors (ARFs) and Ras-like proteins in brain (RABs), in addition to VAMP3, DENND4C, and EXOC7 indicated that IL-2 signaled to molecules that control intracellular vesicle transport and exocytosis (Figure 1H).

IL-2 Regulation of the Nuclear Environment and mRNA Translational Machinery

IL-2 controls transcriptional programs to promote CTL terminal differentiation (Macintyre et al., 2011; Pipkin et al., 2010). In addition to the Tyr phosphorylations that promote STAT5 DNA binding, the present data showed that IL-2 controlled STAT5A S127 and S128 phosphorylation (Figure 2A). IL-2-induced Ser phosphorylation of STAT5 has been proposed to regulate STAT5 transcriptional function (Beadling et al., 1996; Clark et al., 2005). IL-2-regulated phosphoproteins included other transcription factors such as MYC and NFIL3A; proteins that modify histones and chromatin; DNA helicases; and constituents of the RNA polymerase II machinery such as TAF3—a TFIID subunit—and the RNA polymerase II subunit A C-terminal domain phosphatase, CTDP1 (Figure 2A and Table S2). IL-2 reproducibly increased TRIM28 S473 phosphorylation (Figure 2A). TRIM28 is a co-repressor that mediates transcriptional silencing and is important for T cell development and peripheral T cell homeostasis (Chikuma et al., 2012). TRIM28 complexes with heterochromatin

Figure 1. Phosphoproteomic Analysis of IL-2 Maintained CTL

(A) Experimental workflow for SILAC-based quantitative phosphoproteomic analysis of T cells.

(B) CTLs differentiated as in (A) were starved of IL-2 for 24 hr in the presence of IL-12 to sustain expression of CD25 (IL-2 quiesced). The heavy-labeled CTLs were stimulated with 20 ng/mL IL-2 for 15 min mixed with the control (light) cells, and phosphopeptides were prepared. Phosphosites identified in three biological replicates are shown, with log-transformed SILAC ratios plotted against log-transformed p values (one sample t test); refer to Tables S1 and S2 for identified phosphosites. Phosphosites with ratios reproducibly changed by 1.5-fold are shown in dark gray. The 50 phosphosites most reproducibly increased by IL-2 are shown in red and are displayed alongside. Phosphosites found to show a statistically significant regulation (p value $\leq$ 0.05) are marked with an asterisk (*).

(C) The overlap and correlation in the SILAC ratios of the phosphosites identified in the individual biological replicates is shown.

(D) The numbers and percentages of phosphosites regulated in each replicate are shown.

(E) CTL deprived of IL-2 and maintained in IL-12 were treated with or without IL-2 for 15 min and analyzed for STAT5 and ERK phosphorylation by immunoblot.

(F) A schematic representation of the IL-2-regulated phospho-Tyr residues is shown.

(G) The proteins regulated by phosphorylation in response to IL-2 were evaluated for function. The graph shows the cluster enrichment score as determined by DAVID analysis. See also Table S3.

(H) Overview of selected phosphosites regulated consistently by IL-2 in two or more experiments in proteins that regulate the cytoskeleton or vesicle transport.

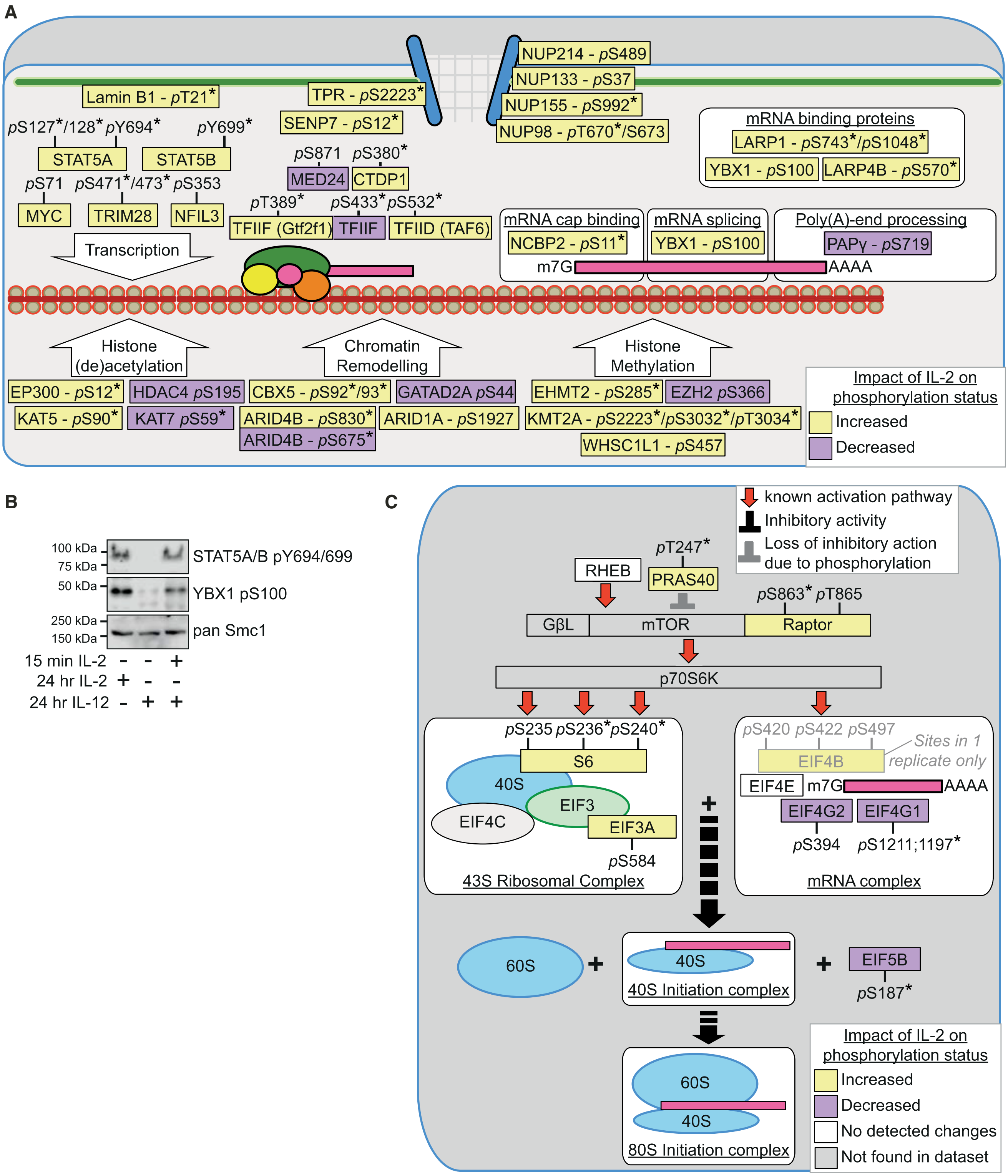

Figure 2. IL-2 Couples the Control of the Nuclear Environment with Translation

(A) Schematic overview of selected phosphosites in nuclear proteins regulated consistently by IL-2 in quiesced CTLs in two or more experiments.

(B) Immunoblot analysis of the phosphorylation of YBX1 in CTLs.

(C) Schematic overview of mTORC1 signaling and translational machinery proteins identified in the IL-2 dataset. Phosphosites found to show a statistically significant regulation (p value $\leq$ 0.05, one sample t test) are marked with an asterisk (*).

See also Table S2.

protein 1 (HP1) family proteins to control chromatin remodeling; the phosphorylation of S473 in TRIM28 inhibits HP1 binding and co-repressor function (Chang et al., 2008). Together, these data expose the extensive influence IL-2 may have on transcription.

Implementation of gene expression relies on mRNA transcript processing and translation. Interestingly, IL-2 stimulated the phosphorylation of nuclear pore proteins, including Nup98 and Nup214, that are required for mRNA export into the cytoplasm (Figure 2A). IL-2 also controlled phosphorylations on proteins that direct RNA stability (Figure 2A). IL-2 stimulated phosphorylation of S100 in YBX1, a component of messenger ribonucleoprotein particles (mRNPs). This phosphorylation was validated using phospho-specific antibodies (Figure 2B). YBX1 binds to mRNAs to prevent their association with the translation initiation complex: phosphorylation of YBX1 on S100 blocks mRNA binding, thereby permitting translation of YBX1 binding mRNAs (Evdokimova et al., 2006).

Thus, the current data argue that IL-2 has the potential to coordinate the composition of the nuclear proteome, the function of the nuclear pore, and the RNA binding capability of mRNPs to orchestrate which gene transcripts are processed into protein in CTLs. Additionally, we noted that IL-2 regulated the phosphorylation of key components of the translational machinery, e.g., EIF5B (Figure 2C), which is critical for translation initiation, including stabilizing the association of the initiation methionine-tRNA with the ribosome and regulating ribosome assembly (Lee et al., 2014). The phosphoproteomic dataset also confirmed previous observations that IL-2 regulates the activity of mTORC1 (Figure 2C), a kinase that controls mRNA translation and protein degradation pathways and shapes the CTL proteome (Hukelmann et al., 2016).

JAK-Controlled Phosphorylation Pathways in CTLs

The Tyr kinases JAK1 and JAK3 are important for IL-2 signaling, but their role in regulating CTL phosphoproteomes has not been defined. Accordingly, we used SILAC-based mass spectrometry to compare the phosphoproteome of CTLs maintained in IL-2 alone both before and after a 30-min or 4-hr treatment with the JAK3 and JAK1 inhibitor, Tofacitinib. The ability of Tofacitinib to inhibit JAKs was verified by its ability to cause a rapid and sustained loss of STAT5 Tyr phosphorylation (Figure 3A). The collective analysis of three biological replicates for the 30-min Tofacitinib treatment identified 8,839 phosphosites on 3,086 proteins (Figure 3B and Table S4). In each replicate, Tofacitinib decreased phosphorylation on ~4% of the identified phosphorylation sites (Figure S1A). It was also striking that Tofacitinib caused an increase in ~4% of the detected phosphoproteome (Figure 3B). Collectively, these experiments identified 283 phosphosites that were decreased and 237 phosphosites that were increased in response to 30 min of Tofacitinib treatment of CTLs (Figure 3B). Following 4 hr of Tofacitinib treatment, we identified 11,822 phosphosites in CTLs from 3,499 proteins (Figure 3C and Table S5). In total, we identified 450 downregulated and 185 upregulated phosphorylations in the 4-hr Tofacitinib-treated CTLs (Figure 3C).

Both datasets confirmed that Tofacitinib treatment caused a rapid and sustained loss of STAT5A and STAT5B Tyr phosphorylation but also revealed a decrease of the IL-2-regulated Tyr phosphorylations in SHC1 (pY313) and GAB3 (pY569) (Figure 3D). The rapidity in the loss of IL-2-regulated Tyr phosphorylations following JAK inhibition is consistent with high levels and/or activity of protein Tyr phosphatases in CTLs. Additionally, Tofacitinib treatment impacted the Ser/Thr phosphorylation network in CTLs; the ratio of pS:pT:pY in the Tofacitinib-regulated phosphosites was 35:6.5:1. Indeed, there were striking changes to a core set of pS and pT phosphorylations at both the 30-min and 4-hr time points (Table S6). Moreover, there was clear reciprocal regulation of Ser/Thr phosphorylation sites modulated by IL-2 and Tofacitinib (Figure 3E), including those in L-plastin, Stathmin, DENND4C, and STAT5. These data indicate that an IL-2-JAK1/3 pathway controlled diverse Ser/Thr kinases in CTLs. In particular, the phosphoproteomics indicated that JAKs couple IL-2Rs to regulation of mRNA stability and translation and, hence, protein synthesis (Figure 3F), as there was reciprocal regulation of the phosphorylation of YBX1, LARP1, LARP4B, and EIF3A by IL-2 and Tofacitinib (Figure 3E). We tested this hypothesis using a sensitive single-cell assay that quantifies the catalytic incorporation of an analog of puromycin, an aminoacyl-tRNA mimetic, into elongating nascent protein chains in the ribosome. IL-2-stimulated CTLs have high protein synthesis and a high protein content compared to CTLs treated with Tofacitinib or CTLs cultured without cytokine (Figures 3G and 3H). The prediction from phosphoproteomics was thus correct: IL-2-JAK signaling pathways are essential for protein synthesis and maintenance of CTL mass.

Evidence for Tofacitinib Insensitive IL-2 Signaling in CTLs

Interestingly, we uncovered some discordance between the IL-2- and Tofacitinib-regulated phosphoproteomes. Notably, IL-2 stimulation triggered dephosphorylation of a number of proteins that did not show increased phosphorylation in response to Tofacitinib inhibition of JAK3 and/or JAK1 (Figures 4A–4C). For instance, Cofilin, an important regulator of actin filament dynamics, is inactivated by S3 phosphorylation and reactivated by S3 de-phosphorylation mediated by phosphatases such as slingshot 1 (SSH1) (Mizuno, 2013). IL-2 stimulated dephosphorylation of Cofilin S3, yet phosphorylation on this site was not controlled by Tofacitinib (Figure 4C). Thus, IL-2 may orchestrate Tofacitinib-independent signaling pathways. It is also possible that there are different thresholds of JAK signaling needed for different responses. The Tofacitinib concentrations used herein totally blocked STAT5 phosphorylation, but it is impossible to exclude a small pool of JAK molecules that were inaccessible to the inhibitor. We also noted that some IL-2-regulated phosphorylations were only lost after sustained Tofacitinib treatment, e.g., STAT5A Y694 was dephosphorylated within 30 min of Tofacitinib treatment, whereas STAT5A S127 and S128 were only decreased in the 4-hr treatment. Protein dephosphorylation following kinase inhibition is determined by the activity of relevant phosphatases. The differential kinetics of STAT5 Tyr and Ser dephosphorylation informs about the relative abundance and/or activity of the STAT5 Tyr and Ser phosphatases. Hence, the finding that a significant subset of IL-2-stimulated phosphorylations was not decreased following 4 hr of Tofacitinib treatment (Figures 4A–4C) could reflect that these are very stable modifications due to low activity and/or accessibility of the relevant phosphatases.

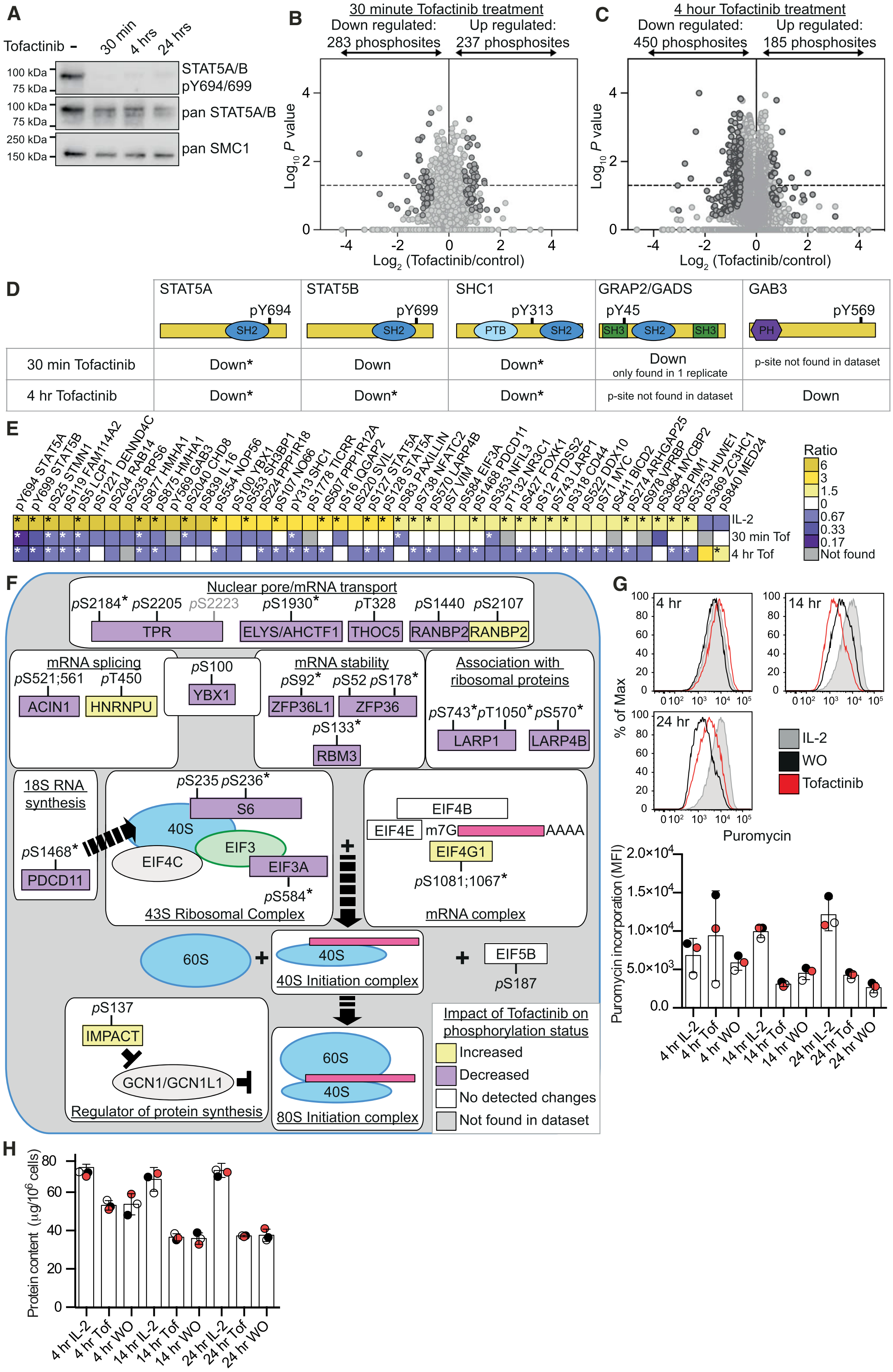

(legend on next page)

IL-2- and JAK-Independent SRC Family Kinase Signaling Networks in CTLs

The phosphoproteomic datasets identified 13,134 phosphosites on 3,706 proteins in CTLs that were not modulated by IL-2 or by Tofacitinib treatment (Table S7). The majority of these were Ser/Thr phosphorylations but included a subset of 105 Tyr phosphorylations in 93 proteins comprising adaptor proteins and enzymes (Figure 4D). Among them were regulatory pY sites in the SRC kinases, LCK (Couture et al., 1996; Marth et al., 1988) and FYN (Maksumova et al., 2005); the Tec family Tyr kinases, TEC (Titz et al., 2010) and TXK (Chamorro et al., 2001); and the pseudokinase SGK223 (Safari et al., 2011) (Figure 4E). Interestingly, the protein Tyr phosphatases, PTPN6/SHP-1 and PTPN11/SHP-2, were phosphorylated on key regulatory sites required for their optimal activity (Cunnick et al., 2002; Lu et al., 2001; Zhang et al., 2003) (Figure 4E). Notably, these Tyr phosphosites showed a strong representation of SRC family kinase consensus motifs and sites experimentally assigned as SRC family kinase substrates (Table S8). These included the regulatory phosphorylation in the activating loop of TXK/RLK (pY420) and the corresponding phosphorylation in TEC (pY518) and pY170 in CD3 epsilon (de Aós et al., 1997). Moreover, we identified two well-characterized SRC substrates in non-lymphoid cells: pY118 in paxillin (Vindis et al., 2004) and pY44 in the metabolic enzyme, enolase (Luo et al., 2008; Tanaka et al., 1995). CTLs co-express the SRC family kinases LCK and FYN, and the phosphoproteomic data revealed that these kinases were phosphorylated on sites associated with catalytic activation: pY394 in LCK and pY420 in FYN (Figure 4E). The SRC family kinase phosphorylation signature, particularly the activating phosphorylations of LCK and FYN, was not regulated by IL-2 or by Tofacitinib. The SRC kinase phosphorylation signature was identified in CTLs maintained in IL-2 alone and also in the quiescent CTLs maintained in IL-12. Moreover, phosphoproteomics analysis revealed that the SRC kinase signature was not increased by IL-12 treatment of IL-2-maintained CTLs (Figure S2A). The data are consistent with previous reports that SRC family kinases are constitutively active in T cells (Nika et al., 2010).

To explore LCK- and/or FYN-controlled phosphorylations in CTLs, we used the selective SRC family kinase inhibitor, PP2. This inhibitor did not prevent IL-2 activation of JAKs, as STAT5A Y694 and STAT5B Y699 phosphorylations were sustained in IL-2-maintained CTLs following exposure to PP2 (Figure 5A). Moreover, prolonged PP2 treatment did not cause loss of expression of CD25, a well-established JAK1/3-STAT5-regulated protein (John et al., 1996; Kim et al., 2001; Lin et al., 2012) (Figure 5B), nor did PP2 mimic the impact of IL-2 withdrawal on CTL size (Figure 5C) or mass (Figure 5D). SILAC-based quantitative mass spectrometry analysis of IL-2-maintained CTLs, both before and after treatment with PP2, confirmed PP2 selectivity. Effective PP2 inhibition of LCK and/or FYN was emphasized by decreased phosphorylation of TXK/RLK pY420, Enolase pY44, paxillin pY118, SGK223 pY196, and PTPRα pY825 (Figure 5E), yet JAK1 and JAK3 signaling remained intact. PP2 treatment neither downregulated STAT5A pY694 and STAT5B pY699 phosphorylation (Figure 5E) nor modulated autophosphorylation sites of JAK1 (pY1033) or JAK3 (pY781). There was also little overlap in the Tyr phosphorylations downregulated by Tofacitinib versus PP2 (Figure 5E). For example, PP2 treatment also did not inhibit SHC1 pY313 Tyr phosphorylation (Figure 5E), a key step in the activation of the Ras-ERK1 and ERK2 pathway. Indeed, PP2 treatment did not inhibit ERK1 and ERK2 phosphorylation in IL-2-stimulated CTLs (Figure 5F).

Collectively, PP2 decreased 779 phosphorylations on 554 proteins and increased 469 phosphorylations on 349 proteins in CTLs (Figures 5G and S2B and Table S9). The limited overlap with the Tofacitinib-regulated phosphorylations (Figure 5H) consisted mainly of pS and pT sites. It was notable that phosphoproteins reproducibly regulated by PP2 were enriched in ATP-binding proteins, including diverse Ser/Thr kinases (Figure 5I and Table S10). This impacted signaling by protein kinase C; protein kinase D2; the MAP kinase, ERK3; and the AMP family kinases SIK1, SIK2, and SIK3 (Figure 5J). The data also indicated that LCK- and/or FYN-mediated signaling restrains the activity of the MAP2K4-p38-MAPKAP2 Ser/Thr kinases (Figure 5J).

There were a small number of phosphorylations that were co-regulated by IL-2-JAK1/3 and SRC kinases (Figure 6A and Table S11). These include mTORC1-controlled phosphorylations on ribosomal S6 proteins (Figures 6A and 6B). Flow cytometric quantification of S6 pS235 and/or pS236 phosphorylations using phospho-specific antibodies confirmed that IL-2-maintained CTLs have high levels of S6 phosphorylation that are downregulated in CTLs treated with either Tofacitinib, PP2, or the mTORC1 inhibitor rapamycin (Figure 6C). Moreover, western blot analysis of the phosphorylation of the mTORC1 substrate sequence on S6K1 (pT389) confirmed that activity of both JAKs and SRC

Figure 3. Analysis of the Impact of the JAK1/JAK3 Inhibitor, Tofacitinib, on the IL-2-Maintained CTL Phosphoproteome

(A) Immunoblot analysis of the STAT5 phosphorylation in CTLs maintained in IL-2 only over a time course of treatment with 100 nM Tofacitinib, a JAK1/3 inhibitor.

(B and C) Heavy-labeled SILAC CTLs were treated with 100 nM Tofacitinib for 30 min (B) or 4 hr (C) before being mixed with control (light) CTLs for phosphoproteome analysis. Log-transformed SILAC ratios are plotted against log-transformed p values (one sample t test). See also Figure S1, and refer to Tables S4 and S5 for lists of phosphopeptides. The graphs in (B) and (C) show the phosphosites identified in three biological replicates. Phosphosites with ratios reproducibly changed by 1.5-fold are shown in dark gray.

(D) The impact of Tofacitinib on the IL-2-regulated Tyr phosphorylation sites is shown.

(E) The heatmap shows phosphosites inversely regulated by IL-2 and Tofacitinib.

(F) Tofacitinib-regulated phosphorylation sites identified in proteins involved in mRNA processing, transport, stability, and translation are shown. See also Table S6.

(D–F) Phosphosites found to show a statistically significant regulation (p value $\leq$ 0.05, one sample t test) are marked with an asterisk (*).

(G) Protein synthesis of CTLs maintained in IL-2 only, or IL-2 maintained CTL treated with 100 nM Tofacitinib (Tof) or deprived of any cytokines (WO) for different times was measured by puromycin incorporation using a flow-cytometry-based assay. Representative flow cytometry plots of the puromycin incorporation measurements are shown, with color-matched individual replicate data from three biological replicates shown alongside. The quantified protein content, as determined by BCA assay, of the same cells at each time point is shown in (H). Bars in (G) and (H) show the mean ± SD.

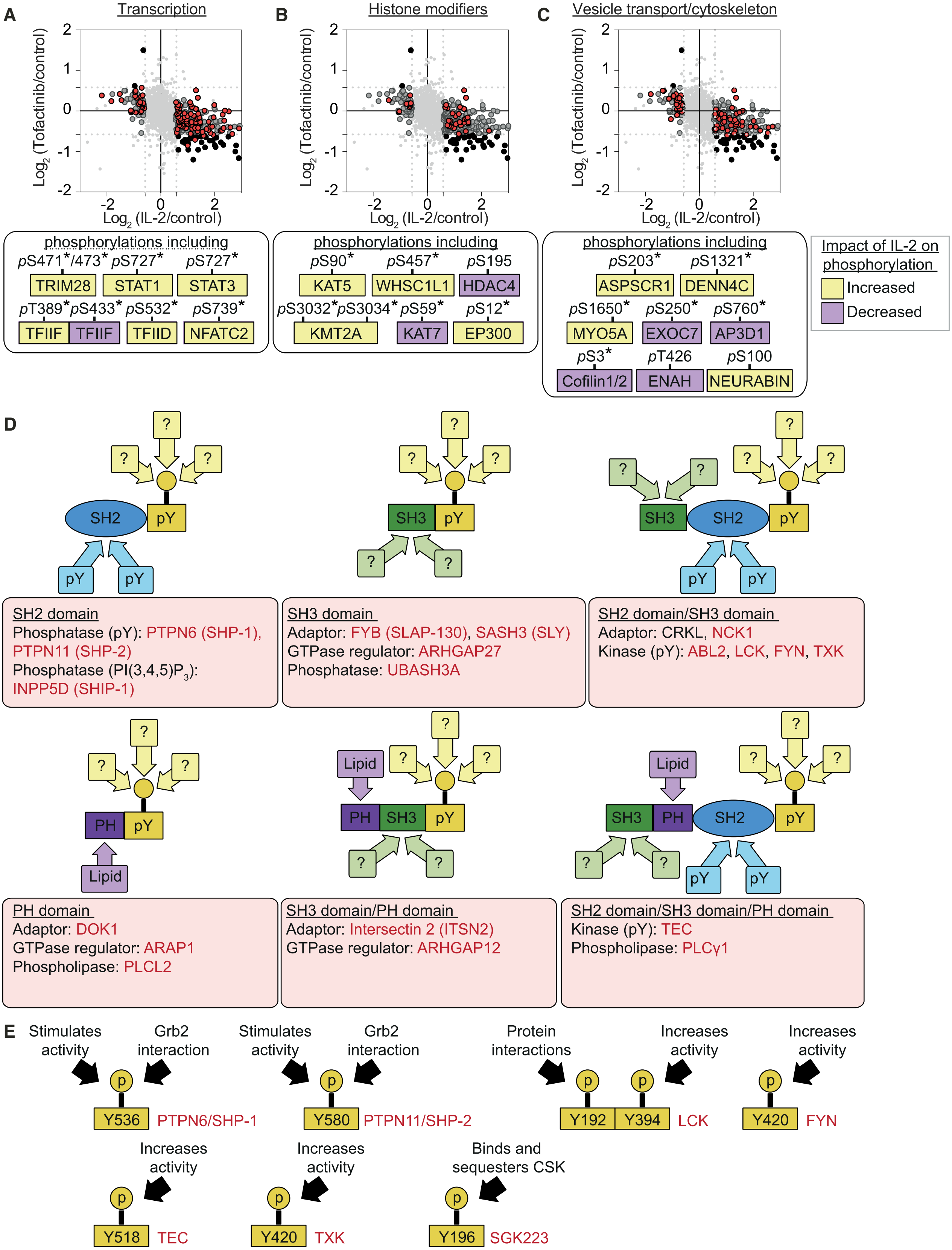

(legend on next page)

kinases is required to sustain mTORC1 activity in CTLs (Figure 6D). Collectively, these data show that IL-2-JAK signaling coordinates with SRC-kinase-controlled phosphorylation pathways to control mTORC1 activity in CTLs.

SRC Family Kinase Regulation of PIP_3-AKT Signaling Pathways in CTLs

The Ser/Thr kinase AKT plays an important role in CTLs to regulate nuclear exclusion and function of the FOXO1 and FOXO3 transcription factors. These simultaneously induce and repress expression of genes encoding key effector and trafficking molecules to direct effector and/or memory $CD8^+$ T cell differentiation (Hedrick et al., 2012). It has been proposed that the PI3K-AKT-mTORC1 pathway is sensitive to JAK3 inhibition (Smith et al., 2016). However, this conclusion is based on experiments with a new JAK3 inhibitor that monitored S6 phosphorylation as a surrogate for AKT activity (Smith et al., 2016). The present data show that mTORC1-mediated phosphorylation of S6 in CTLs is also Tofacitinib sensitive (Figures 6C and 6D). However, a salient fact is that the activity of mTORC1 and the phosphorylation of S6 are independent of AKT or PI3K in CTLs (Finlay et al., 2012; Hukelmann et al., 2016). Therefore, to assess the effect of JAK inhibitors on AKT activity, more direct assays and analyses are required.

The current mass spectrometry dataset had little coverage of AKT or AKT phosphosites reflecting biases against the detection of the R/K-X-R/K-X-X-pS/T-φ AKT substrate sequence when using trypsin for protein digestion (Giansanti et al., 2015). We did detect one putative AKT substrate sequence, PRAS40/AKT1S1 T247 phosphorylation, which was increased following IL-2 stimulation but was neither modulated by Tofacitinib nor by PP2 treatment (Figure 7A).

To explore the role of IL-2 and JAKs in controlling AKT activity in more detail, we therefore adopted biochemical experiments. The activity of AKT is controlled by PDK1-mediated phosphorylation of T308 in the AKT catalytic domain. The association between AKT and PDK1 is facilitated both by PIP_3 and the mTORC2-mediated phosphorylation of AKT on S473 (Najafov et al., 2012). Accordingly, we directly assessed the regulation of AKT phosphorylation by JAK and SRC kinase pathways. IL-2-cultured CTLs had high AKT T308 and S473 phosphorylation, which was lost if cells were treated with the PI3K-p110δ inhibitor, IC87114, or the allosteric inhibitor, AKTi, which prevents PIP_3 binding to AKT (Figure 7B). These experiments showed that AKT activity in CTLs was dependent on sustained production of PIP_3 and sustained interaction of PIP_3 and AKT. CTLs treated with Tofacitinib lost STAT5 Tyr phosphorylation and decreased phosphorylation of AKT S473 (Figure 7B), but there was only a minimal effect of Tofacitinib on AKT T308 phosphorylation and no detectable effect of Tofacitinib on the phosphorylation of the FOXO1 pT24 and FOXO3 pT32 AKT substrate sequences (Figure 7B). However, PP2 blocked the phosphorylation of AKT T308 and FOXO1 pT24 and/or FOXO3 pT32 (Figure 7B). We also found that IL-2 deprivation for 60 min resulted in complete loss of STAT5, but not AKT phosphorylation (Figure 7C). Hence, SRC kinases pathways, rather than IL-2-JAK signaling, controlled AKT in CTLs. One role for AKT is to cause phosphorylation-mediated nuclear exclusion of FOXO1. We used two strategies to examine the impact of JAK and SRC kinases on the localization of FOXO1 in CTLs: microscopic analysis of intact cells and flow cytometry analysis of purified nuclei from CTLs expressing a FOXO1-GFP fusion protein. In IL-2-maintained CTLs, which have high AKT activity, FOXO1-GFP was predominantly localized to the cytoplasm (Figures 7D and 7E). When CTLs were treated with IC87114 (Figure 7F) or AKTi (Figure 7G), the FOXO1-GFP relocated to the nucleus. The majority of FOXO1-GFP also relocalized to the nucleus in PP2, but not Tofacitinib-treated, CTLs (Figures 7H–7J).

As PIP_3 levels are rate limiting for AKT activation, the differential sensitivity of AKT phosphorylation and activity to PP2 and Tofacitinib raised the possibility that SRC kinases, rather than JAK signaling, controlled PIP_3 levels in IL-2-maintained CTLs. Lipid measurements confirmed that PIP_3 decreased in CTLs following PI3K-p110δ inhibition and PP2 treatment. However, there was no discernible effect of Tofacitinib on PIP_3 levels in CTLs (Figure 7K). Importantly, cellular levels of phosphatidylinositol (4,5)-bisphosphate (PIP_2), the precursor of PIP_3, were not changed by the inhibitors (Figure S3). We also investigated the IL-2 dependence of PIP_3 production and noted that neither PIP_3 levels nor AKT phosphorylation rapidly declined in IL-2-deprived CTLs (Figures 7K and 7L). Thus, IL-2R occupancy is not tightly coupled to PIP_3 production, AKT activation, or FOXO1 phosphorylation and nuclear exclusion in CTLs. Rather, our data support a model where IL-2-JAK signaling integrates with IL-2-JAK-independent phosphorylation networks to program CTL fate (Figure S3).

DISCUSSION

This study provides a systematic characterization and extensive documentation of the IL-2-regulated phosphoproteome and an SRC-family-kinase-controlled phosphorylation network in cytotoxic T cells. Our data afford new perspectives about the diverse Ser/Thr phosphorylations controlled by IL-2 to direct T cell biology and afford novel insights into critical signaling networks that can modify the outcome of IL-2 signaling and control CTL fate. We found that SRC family kinases regulated a substantial component of the CTL phosphoproteome and regulated

Figure 4. The IL-2-Dependent and -Independent CTL Phosphoproteome

(A–C) Average SILAC ratios for 4 hr Tofacitinib treated CTLs maintained in IL-2 only were plotted against average SILAC ratios for IL-2-stimulated quiescent (starved of IL-2 but maintained with IL-12) CTLs. All data are shown in light gray, with dark gray circles indicating the phosphorylation sites regulated by IL-2, but not by Tofacitinib; the black circles show the phosphorylation sites inversely regulated by IL-2 and Tofacitinib; and the red points showing the phosphosites on proteins grouped by their annotated GO functions in transcription (A), histone modification (B), or vesicle transport and the cytoskeleton (C). Selected phosphorylation sites regulated by IL-2, but not Tofacitinib, are shown below each graph.

(D) Schematic representation of selected Tyr phosphorylated proteins identified in quiescent and IL-2 maintained CTL that are not regulated by IL-2 or JAK1/3 inhibition. Where identified phospho-Tyr residues fit a SRC family kinase motif, the protein is shown in red. See also Table S8.

(E) The location and function of certain identified Tyr phosphorylation sites is shown.

CellPress

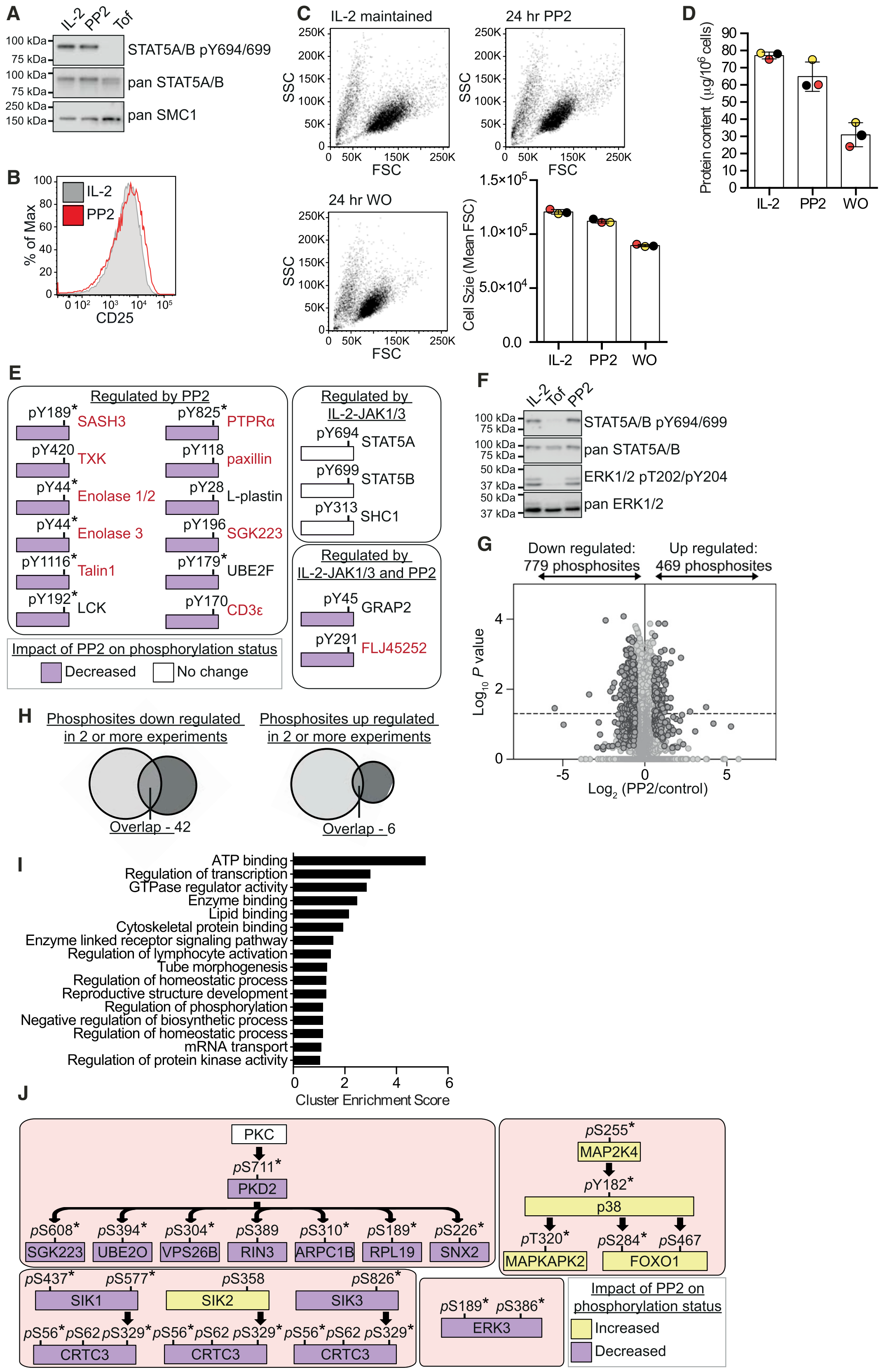

(legend on next page)

CellPress

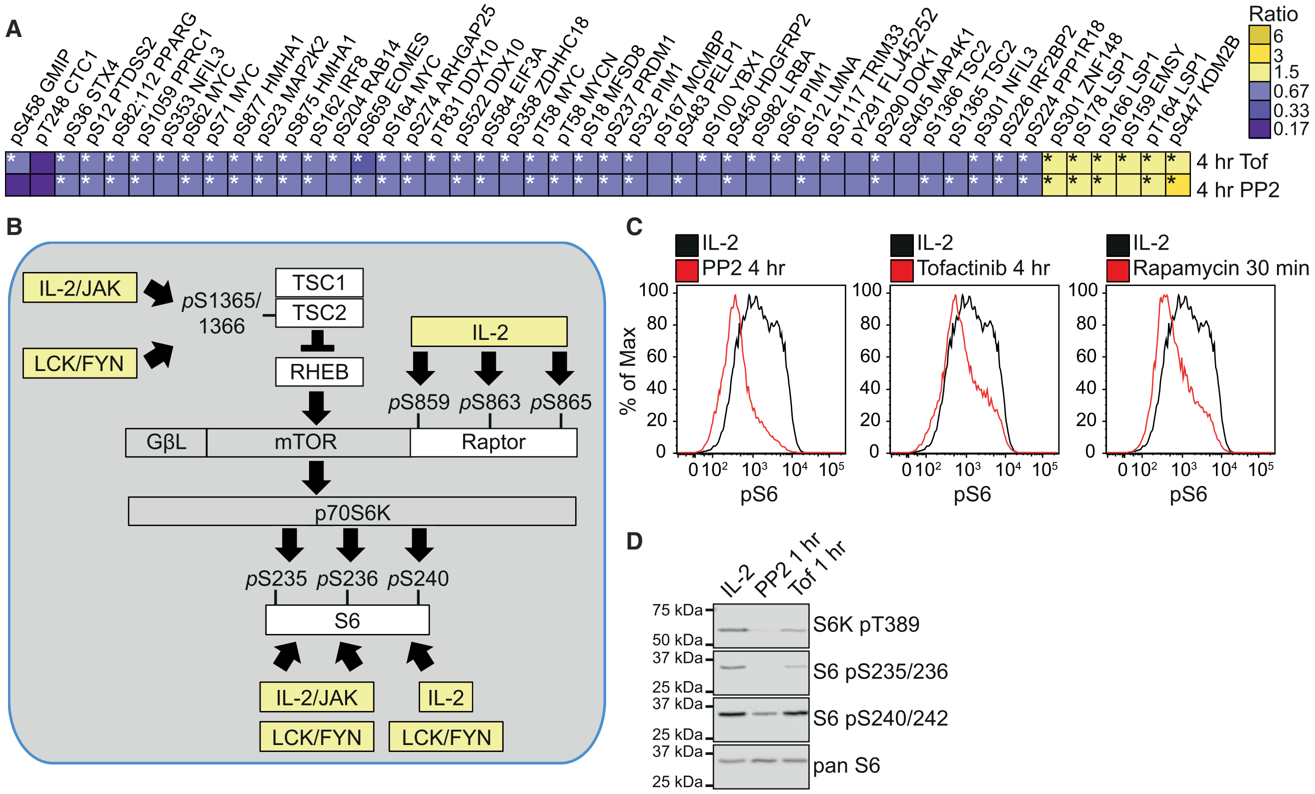

Figure 6. Comparison of Tofacitinib- and PP2-Co-regulated Phosphosites in CTL

(A) The heat map shows phosphosites residues reproducibly identified and co-regulated after 4 hr of Tofacitinib (Tof) or 4 hr of PP2 treatment in CTL maintained in IL-2 alone. Phosphosites found to show a statistically significant regulation in each condition (p value $\leq$ 0.05, one sample t test) are marked with an asterisk (*). See also Table S11.

(B) Schematic representation of the mTORC1 pathway and the regulation of the phosphorylation of components by IL-2-JAK and LCK and/or FYN signaling, as determined by the phosphoproteomic analyses.

(C) The phosphorylation status of S6, which is phosphorylated when mTORC1 is active, was quantified in CTLs maintained in IL-2 alone and treated with 100 nM Tofacitinib, 10 μM PP2, and 20 nM Rapamycin, the mTORC1 inhibitor, using a phospho-specific antibody and flow cytometry.

(D) The phosphorylation status of the mTORC1 substrate, S6K (pT389) and its substrate S6 (pS235, pS236 and pS240, pS242) was determined in IL-2-maintained CTL by immunoblot. The data in (C) and (D) are representative of at least three experiments.

phosphorylations that were distinct from those modulated by IL-2-JAK1/3. Links between SRC kinases and IL-2 signaling were first described 25 years ago (Hatakeyama et al., 1991; Horak et al., 1991; Kobayashi et al., 1993; Zhou et al., 2000). We found no evidence that IL-2-JAK signaling stimulated LCK and/or FYN kinase activity; rather, a pool of active SRC family kinases was required to sustain the activity of critical Ser/Thr kinases, such as mTORC1 and AKT, in IL-2-stimulated CTLs.

Figure 5. Impact of the SRC Kinase Inhibitor, PP2, on the CTL Phosphoproteome

(A) Immunoblot analysis of STAT5 pY phosphorylation in CTLs maintained in IL-2 only and treated with 10 μM PP2 or 100 nM Tofacitinib (Tof) for 1 hr.

(B) Flow cytometry analysis of the expression of CD25 in IL-2-maintained CTLs treated without or with 10 μM PP2 for 24 hr. The histogram is representative of at least three experiments.

(C and D) CTLs differentiated in IL-2 alone were treated with 10 μM PP2 or deprived of IL-2 (WO) for 24 hr, and the size of the cells was evaluated by flow cytometry and compared to IL-2-maintained controls. Representative FSC and SSC profiles are shown in (C), with mean FSC values of three color-matched biological replicates shown alongside. The protein content of the same cells was determined by BCA assay and is shown in (D). In (C) and (D), the bars show the mean ± SD.

(E and G–J) Heavy-labeled SILAC CTLs differentiated and maintained in IL-2 alone were treated with 10 μM PP2 for 4 hr before being mixed with control (light) cells for phosphoproteome analysis. The regulated Tyr phosphorylation sites identified in the dataset were compared to those regulated by IL-2 and/or Tofacitinib (E). Protein names are shown in red when the identified phosphorylation site fits a SRC family kinase motif or is a known SRC family kinase substrate.

(F) The immunoblot analysis shows the impact of a 30-min treatment of 100 nM Tofacitinib (Tof) or 10 μM PP2 on ERK phosphorylation in CTL maintained only in IL-2.

(G) An overview of all the phosphosites identified in three biological replicates is displayed. Phosphosites with ratios reproducibly changed by 1.5-fold are shown in dark gray. See also Figure S2 and refer to Tables S9 and S10 for full lists.

(H) Phosphosites regulated by 4 hr PP2 or 4 hr Tofacitinib treatment were evaluated for overlap.

(I) The proteins with phosphorylation sites reproducibly regulated by 4 hr of PP2 treatment were evaluated for function. The cluster enrichment score as determined by DAVID analysis is shown.

(J) Schematic representations of selected pS and pT signaling pathways perturbed by PP2 are shown in (J). In (E) and (J), phosphosites found to show a statistically significant regulation (p value $\leq$ 0.05, one sample t test) are marked with an asterisk (*).

A

	IL-2/control			Tofact/cont			PP2/control		
	Expt1	Expt2	Expt3	Expt1	Expt2	Expt3	Expt1	Expt2	Expt3
T247 Akt1s1	1.70	2.00	1.98	0.82	0.73	0.77	1.02	0.87	0.99

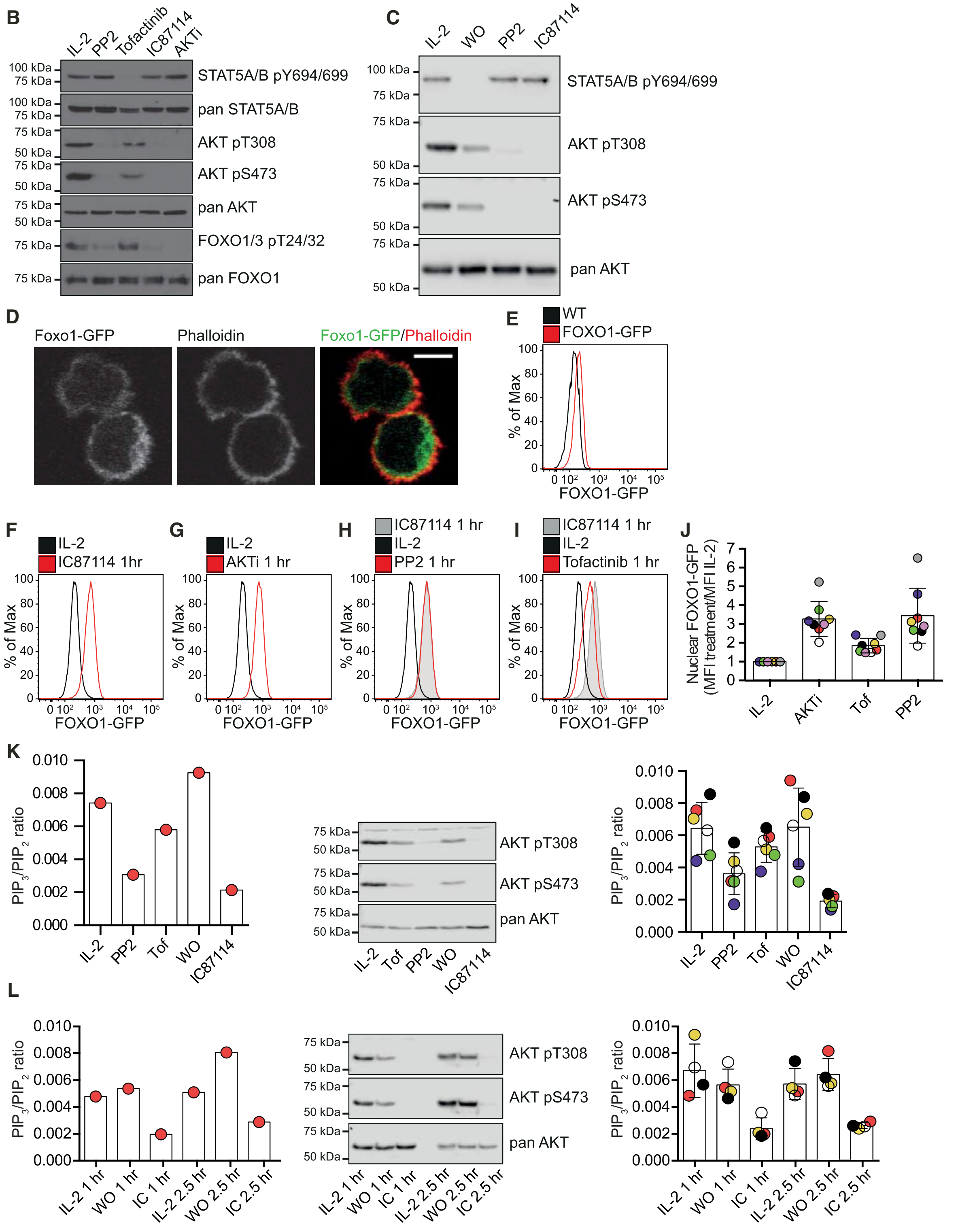

(legend on next page)

One new insight from our data was that IL-2-JAK signaling had a complex impact on the nuclear environment of T cells beyond STAT5 nuclear translocation. The IL-2-JAK-regulated CTL phosphoproteome was dominated by proteins that regulate RNA, the protein translational machinery, vesicular trafficking, exocytosis, and the cytoskeleton. This diversity affords an explanation for the broad role of IL-2 as a regulator of the T cell biology. In particular, we noted that JAK activity linked the IL-2 receptor to regulatory phosphorylations on proteins that control mRNA stability and translation. This prompted the experiments that revealed that IL-2 sustained mRNA translation and protein synthesis in T cells. Thus, IL-2 can configure T cell proteomes independently of the transcriptional program. The ability of IL-2 to control rates of protein synthesis is a mechanism to explain the documented discordance between the transcriptome and proteome of IL-2-maintained CTLs (Hukelmann et al., 2016). Moreover, our results highlight that understanding how to manipulate IL-2 signaling for therapy needs to consider how IL-2 controls the biosynthetic capacity of the cell. The complexity of IL-2 signaling pathways in CTLs revealed by our data demonstrates the need for future studies that address the kinetics of phosphorylation events and determine if these pathways are always coordinately regulated or whether different thresholds of IL-2R occupancy activate different IL-2 signaling nodes.

Another important new perspective identified in this study was the relationship between IL-2R occupancy, JAK activity, and the control of PIP_3-AKT signaling. Understanding how T cells regulate PIP_3-AKT signaling is important, as the strength and coordination of AKT activity is pivotal for effector T cell differentiation (Macintyre et al., 2011) and because immune function is impaired both by constitutive activation and by loss of PI3K-p110δ activity (Angulo et al., 2013; Lucas et al., 2014; Okkenhaug et al., 2007). Current IL-2 signal transduction models place PIP_3-AKT pathways downstream of JAKs. Our data force a revision of this model, as we found that, in CTL, the PIP_3-AKT pathway was regulated by JAK-independent SRC-family-kinase-mediated signaling. The PP2 sensitivity of cellular PIP_3-AKT signaling in CTLs suggests that IL-2-JAK signaling controls CTL function by integrating with pathways of lipid signaling and protein phosphorylation that are organized prior to IL-2R occupancy. The significance of “pre-organized” pathways of protein phosphorylation in T cells is increasingly recognized. For example, LCK activity is essential for antigen receptor signaling in T cells, yet LCK activity is constitutive and not controlled by antigen receptor occupancy (Nika et al., 2010). Likewise, Myc expression in IL-2-maintained CTLs is defined by the constitutive activity of the Ser/Thr kinase GSK3 (Preston et al., 2015), and the constitutive activity of HDAC7 Ser/Thr kinases ensures the nuclear exclusion of this key chromatin regulator in T cells (Navarro et al., 2011). The integration of IL-2-JAK signaling with pre-existing phosphorylation pathways may be a key determinant of the outcome of IL-2R occupancy and may explain how IL-2 can have pleotropic effects in different T cell populations. In particular, if the ability to activate AKT requires the activity of SRC family kinases, and not IL-2 regulated JAKs, our data offer an explanation for the failure of IL-2 to activate AKT in all T cells (Bensinger et al., 2004).

In summary, our data uncovered amazing complexity of protein phosphorylation in CTLs. It revealed a dominance of Ser/Thr phosphorylations, but here, a proviso is that shotgun phosphoproteomics strategies can limit the detection of low-abundance Tyr phosphorylations. Indeed, our dataset did not detect Tyr phosphorylations of IL-2Rβ subunits or JAK1 and JAK3 previously mapped using phospho-Tyr enrichment protocols in the IL-2 dependent cell line, KIT225 (Arneja et al., 2014; Osinalde et al., 2011, 2015). It is also important to note that peptide identifications using SILAC require that proteolytic digests produce phosphopeptides of optimal size for mass spectrometry sequencing (Giansanti et al., 2015). These must also contain an arginine or lysine, as these are the residues that allow phosphopeptide quantification in SILAC-based phosphoproteomics. The development of new mass spectrometry technologies and approaches may allow these limitations to be bypassed. Thus, future screens may produce further insights into signaling in CTL. Nevertheless, collectively, our study mapped over 18,000 phosphorylations in CTL, revealing new insights into the phosphorylation networks that direct the biology of these effector cells and providing a resource for further analyses and discovery.

EXPERIMENTAL PROCEDURES

Mice

Mice were maintained in compliance with UK Home Office Animals (Scientific Procedures) Act 1986 in the University of Dundee. P14 T cell receptor transgenic mice (Pircher et al., 1989) and FOXO1-GFP mice, where GFP was fused to the C terminus of the endogenous FOXO1 gene (Stone et al., 2015), have been described.

Figure 7. Analysis of the PI 3-Kinase-AKT Signaling Pathway in CTL

(A) The regulation of PRAS40/AKT1S1 in CTLs as determined by SILAC phosphoproteomics.

(B and C) Immunoblot analysis of AKT phosphorylation in CTLs maintained in IL-2 alone and treated for 1 hr with 10 μM PP2, 100 nM Tofacitinib, 10 μM IC87114, a PI3K-p110δ inhibitor, or 1 μM AKTi, an AKT inhibitor (B) and in response to 1 hr IL-2 deprivation (C).

(D–J) IL-2-maintained CTLs were generated from FOXO1-GFP mice. The localization of FOXO1-GFP (green) was visualized by microscopy (D), with the actin cytoskeleton detected using phalloidin (red). The scale bar is 5 μm. FOXO1-GFP levels in nuclear extracts of IL-2-maintained CTLs were quantified by flow cytometry under basal conditions (IL-2) and compared to a non-FOXO1-GFP-expressing CTL (WT) (E) or following 1 hr treatment with IC87114 (F), AKTi (G), PP2 (H), or Tofacitinib (Tof) (I). Representative flow cytometry profiles are shown in (F–I), with the level of FOXO1-GFP in each condition expressed as a ratio of the MFI of the IL-2 control of eight color-matched biological replicate treatments shown in (J), with bars showing the mean ± SD.

(K and L) The levels of PIP_3 in CTLs maintained in IL-2 only, shown as a ratio of the measured PIP_2, were determined using mass spectrometry. In (K), IL-2-maintained CTLs were treated with 100 nM Tofacitinib (Tof), 10 μM PP2, 10 μM IC87114, or deprived of IL-2 (WO) for 30 min and compared to IL-2-maintained CTL control cells. In (L), PIP_3 levels were measured in CTL maintained in medium alone (WO) and were compared to control (IL-2) and IL-2 + IC87114-treated cells for up to 2.5 hr. In (K) and (L), the left-hand panels show the data for a single experiment, with the immunoblot analysis of the phosphorylation of AKT on pT308 and pS473 shown in the middle panel. The right panel shows the results for color-matched biological replicates, 6 in (K) and 4 in (L). Bars show the mean ± SD.

Cell Culture and SILAC Labeling

Cytotoxic T Lymphocytes were generated as previously described (Hukelmann et al., 2016; Navarro et al., 2011, 2014) and expanded in RPMI 1640 medium (Life Technologies) supplemented with 10% FBS (Life Technologies), 50 units/mL penicillin-G, 50 μg/mL streptomycin, and 50 μM β-mercaptoethanol and in the presence of 20 ng/mL IL-2 alone (Proleukin, Novartis). For SILAC labeling, CTLs were cultured for 5 days in SILAC RPMI 1640 medium (Life Technologies), supplemented with 200 mg/L L-proline, 84 mg/L L-arginine, 300 mg/L L-glutamate, 10% dialyzed FBS with a 10 kDa cutoff (Thermo Scientific), 50 units/mL penicillin-G, 50 μg/mL streptomycin, 50 μM β-mercaptoethanol, and 20 ng/mL IL-2. The "light" SILAC media contained L-[12C6, 14N4] arginine (R0) and L-[12C6, 14N2]lysine (K0). The "heavy" media contained L-[13C6, 15N4]arginine (R10) and L-[13C6, 15N2]lysine (K8).

For the IL-2 stimulation of CTLs, cells were "IL-2 quiesced" by the removal of IL-2 for 24 hr, but they were supplemented with 20 ng/ml IL-12 (R&D Systems) to sustain cell viability (at ~90%) and the expression of the IL-2Rα chain (CD25). For the Tofacitinib and PP2 studies, CTLs were maintained only in the presence of IL-2, and no IL-12 was added to the culture.

Phosphoproteome Analysis

Sample preparation was performed as described in the Supplemental Experimental Procedures. Three independent biological replicate treatments were performed for each phosphoproteome analysis. The resulting mass spectrometry data were processed using MaxQuant version 1.5.0.0 (Cox and Mann, 2008) and mapped to the reviewed UniProtKB-Swiss-Prot mouse protein database. The output from MaxQuant was filtered to remove known contaminants and reverse sequences before analysis. The distribution of SILAC ratios was normalized within MaxQuant at the peptide level so that the median of Log_2 ratios was zero. Perseus software was used to annotate phosphosites, and the clustering tool in DAVID bioinformatics resources was used for functional annotation.

Flow Cytometry Analysis of Nuclear Extracts

Cells (1×10^6) were treated with 300 μL ice-cold nuclear extraction buffer (3.8 mM trisodium citrate, 9.6 mM NaCl, 0.05% NP-40), and the resulting nuclei were fixed immediately with ice-cold 300 μL IC fixation buffer (eBiosciences) for 15 min at 4°C. After washing, nuclei were stained with DAPI and analyzed using a fluorescence-activated cell sorting (FACS) LSRFortessa flow cytometer with DIVA software (BD Biosciences). Data analysis was performed with FlowJo software (Treestar).

Analysis of Protein Synthesis and Cellular Protein Mass

Cells were treated with O-propargyl-puromycin (OPP, Jena Bioscience) for 10 min, and the incorporation of the aminoacyl-tRNA mimetic into newly synthesized polypeptides was measured by labeling the OPP was with Alexa 647-azide (Invitrogen) using a standard Click-IT chemistry reaction (Invitrogen). Cells were analyzed using a FACSVerse flow cytometer with FACSuite software (BD Biosciences) and analyzed with FlowJo software (Treestar). The protein mass of cells following different treatments was determined by BCA assay as per manufacturer's instructions (Pierce).

Mass Spectrometry Measurements of Inositol Lipids

Inositol lipid measurements were performed by mass spectrometry using 1×10^6 cells per sample as described in the Supplemental Experimental Procedures.

ACCESSION NUMBERS

The mass spectrometry phosphoproteomics data have been deposited to the ProteomeXchange Consortium (http://proteomecentral.proteomexchange.org) via the PRIDE repository with the dataset identifier identifiers PXD004645 and PXD004644.

SUPPLEMENTAL INFORMATION

Supplemental Information contains Supplemental Experimental Procedures, three figures, and eleven tables and can be found online at http://dx.doi.org/10.1016/j.immuni.2016.07.022.

AUTHOR CONTRIBUTIONS

S.H.R. designed, performed, and analyzed most experiments; C.R. performed experiments and provided intellectual input; K.E.A. measured PIP_3; P.T.H. and L.R.S. designed protocols for PIP_3 measurements; D.A.C. and S.H.R. designed the project and wrote the manuscript.

ACKNOWLEDGMENTS

We acknowledge members of the Cantrell group for critical data discussion. We thank the Biological Resources unit, flow cytometry (A. Whigham and R. Clarke) and Proteomics (D. Lamont and team) at the University of Dundee. We thank D. Bensaddek, A. Lamond, R. Gourlay, and M. Trost for assistance with phosphoproteomic protocols. Work was supported by the Wellcome Trust (Principal Research Fellowship 097418/Z/11/Z to D.A.C.) and Tenovus Scotland (S.H.R.). C.R. is the recipient of a studentship from the Biotechnology and Biological Sciences Research Council (BBSRC) and GlaxoSmithKline.

Received: March 7, 2016
Revised: May 3, 2016
Accepted: July 8, 2016
Published: August 23, 2016

REFERENCES

Angulo, I., Vadas, O., Garçon, F., Banham-Hall, E., Plagnol, V., Leahy, T.R., Baxendale, H., Coulter, T., Curtis, J., Wu, C., et al. (2013). Phosphoinositide 3-kinase δ gene mutation predisposes to respiratory infection and airway damage. Science *342*, 866–871.

Arenas-Ramirez, N., Woytschak, J., and Boyman, O. (2015). Interleukin-2: biology, design and application. Trends Immunol. *36*, 763–777.

Arneja, A., Johnson, H., Gabrovsek, L., Lauffenburger, D.A., and White, F.M. (2014). Qualitatively different T cell phenotypic responses to IL-2 versus IL-15 are unified by identical dependences on receptor signal strength and duration. J. Immunol. *192*, 123–135.

Beadling, C., Ng, J., Babbage, J.W., and Cantrell, D.A. (1996). Interleukin-2 activation of STAT5 requires the convergent action of tyrosine kinases and a serine/threonine kinase pathway distinct from the Raf1/ERK2 MAP kinase pathway. EMBO J. *15*, 1902–1913.

Bensinger, S.J., Walsh, P.T., Zhang, J., Carroll, M., Parsons, R., Rathmell, J.C., Thompson, C.B., Burchill, M.A., Farrar, M.A., and Turka, L.A. (2004). Distinct IL-2 receptor signaling pattern in CD4+CD25+ regulatory T cells. J. Immunol. *172*, 5287–5296.

Chamorro, M., Czar, M.J., Debnath, J., Cheng, G., Lenardo, M.J., Varmus, H.E., and Schwartzberg, P.L. (2001). Requirements for activation and RAFT localization of the T-lymphocyte kinase Rlk/Txk. BMC Immunol. *2*, 3.

Chang, C.-W., Chou, H.-Y., Lin, Y.-S., Huang, K.-H., Chang, C.-J., Hsu, T.-C., and Lee, S.-C. (2008). Phosphorylation at Ser473 regulates heterochromatin protein 1 binding and corepressor function of TIF1beta/KAP1. BMC Mol. Biol. *9*, 61.

Chang, V.T., Fernandes, R.A., Ganzinger, K.A., Lee, S.F., Siebold, C., McColl, J., Jönsson, P., Palayret, M., Harlos, K., Coles, C.H., et al. (2016). Initiation of T cell signaling by CD45 segregation at 'close contacts'. Nat. Immunol. *17*, 574–582.

Chikuma, S., Suita, N., Okazaki, I.-M., Shibayama, S., and Honjo, T. (2012). TRIM28 prevents autoinflammatory T cell development in vivo. Nat. Immunol. *13*, 596–603.

Clark, D.E., Williams, C.C., Duplessis, T.T., Moring, K.L., Notwick, A.R., Long, W., Lane, W.S., Beuvink, I., Hynes, N.E., and Jones, F.E. (2005). ERBB4/HER4 potentiates STAT5A transcriptional activity by regulating novel STAT5A serine phosphorylation events. J. Biol. Chem. *280*, 24175–24180.

Cornish, G.H., Sinclair, L.V., and Cantrell, D.A. (2006). Differential regulation of T-cell growth by IL-2 and IL-15. Blood *108*, 600–608.

Couture, C., Songyang, Z., Jascur, T., Williams, S., Tailor, P., Cantley, L.C., and Mustelin, T. (1996). Regulation of the Lck SH2 domain by tyrosine phosphorylation. J. Biol. Chem. *271*, 24880–24884.

Cox, J., and Mann, M. (2008). MaxQuant enables high peptide identification rates, individualized p.p.b.-range mass accuracies and proteome-wide protein quantification. Nat. Biotechnol. *26*, 1367–1372.

Cunnick, J.M., Meng, S., Ren, Y., Desponts, C., Wang, H.-G., Djeu, J.Y., and Wu, J. (2002). Regulation of the mitogen-activated protein kinase signaling pathway by SHP2. J. Biol. Chem. *277*, 9498–9504.

de Aós, I., Metzger, M.H., Exley, M., Dahl, C.E., Misra, S., Zheng, D., Varticovski, L., Terhorst, C., and Sancho, J. (1997). Tyrosine phosphorylation of the CD3-epsilon subunit of the T cell antigen receptor mediates enhanced association with phosphatidylinositol 3-kinase in Jurkat T cells. J. Biol. Chem. *272*, 25310–25318.

Evdokimova, V., Ruzanov, P., Anglesio, M.S., Sorokin, A.V., Ovchinnikov, L.P., Buckley, J., Triche, T.J., Sonenberg, N., and Sorensen, P.H.B. (2006). Akt-mediated YB-1 phosphorylation activates translation of silent mRNA species. Mol. Cell. Biol. *26*, 277–292.

Finlay, D.K., Rosenzweig, E., Sinclair, L.V., Feijoo-Carnero, C., Hukelmann, J.L., Rolf, J., Panteleyev, A.A., Okkenhaug, K., and Cantrell, D.A. (2012). PDK1 regulation of mTOR and hypoxia-inducible factor 1 integrate metabolism and migration of CD8+ T cells. J. Exp. Med. *209*, 2441–2453.

Giansanti, P., Aye, T.T., van den Toorn, H., Peng, M., van Breukelen, B., and Heck, A.J.R. (2015). An augmented multiple-protease-based human phosphopeptide atlas. Cell Rep. *11*, 1834–1843.

Hatakeyama, M., Kono, T., Kobayashi, N., Kawahara, A., Levin, S.D., Perlmutter, R.M., and Taniguchi, T. (1991). Interaction of the IL-2 receptor with the src-family kinase p56lck: identification of novel intermolecular association. Science *252*, 1523–1528.

Hedrick, S.M., Hess Michelini, R., Doedens, A.L., Goldrath, A.W., and Stone, E.L. (2012). FOXO transcription factors throughout T cell biology. Nat. Rev. Immunol. *12*, 649–661.

Horak, I.D., Gress, R.E., Lucas, P.J., Horak, E.M., Waldmann, T.A., and Bolen, J.B. (1991). T-lymphocyte interleukin 2-dependent tyrosine protein kinase signal transduction involves the activation of p56lck. Proc. Natl. Acad. Sci. USA *88*, 1996–2000.

Hukelmann, J.L., Anderson, K.E., Sinclair, L.V., Grzes, K.M., Murillo, A.B., Hawkins, P.T., Stephens, L.R., Lamond, A.I., and Cantrell, D.A. (2016). The cytotoxic T cell proteome and its shaping by the kinase mTOR. Nat. Immunol. *17*, 104–112.

John, S., Robbins, C.M., and Leonard, W.J. (1996). An IL-2 response element in the human IL-2 receptor alpha chain promoter is a composite element that binds Stat5, Elf-1, HMG-I(Y) and a GATA family protein. EMBO J. *15*, 5627–5635.

Kim, H.P., Kelly, J., and Leonard, W.J. (2001). The basis for IL-2-induced IL-2 receptor alpha chain gene regulation: importance of two widely separated IL-2 response elements. Immunity *15*, 159–172.

Kobayashi, N., Kono, T., Hatakeyama, M., Minami, Y., Miyazaki, T., Perlmutter, R.M., and Taniguchi, T. (1993). Functional coupling of the src-family protein tyrosine kinases p59fyn and p53/56lyn with the interleukin 2 receptor: implications for redundancy and pleiotropism in cytokine signal transduction. Proc. Natl. Acad. Sci. USA *90*, 4201–4205.

Lee, S., Truesdell, S.S., Bukhari, S.I.A., Lee, J.H., LeTonqueze, O., and Vasudevan, S. (2014). Upregulation of eIF5B controls cell-cycle arrest and specific developmental stages. Proc. Natl. Acad. Sci. USA *111*, E4315–E4322.

Liao, W., Lin, J.-X., and Leonard, W.J. (2013). Interleukin-2 at the crossroads of effector responses, tolerance, and immunotherapy. Immunity *38*, 13–25.

Lin, J.-X., Li, P., Liu, D., Jin, H.-T., He, J., Ata Ur Rasheed, M., Rochman, Y., Wang, L., Cui, K., Liu, C., et al. (2012). Critical Role of STAT5 transcription factor tetramerization for cytokine responses and normal immune function. Immunity *36*, 586–599.

Lu, W., Gong, D., Bar-Sagi, D., and Cole, P.A. (2001). Site-specific incorporation of a phosphotyrosine mimetic reveals a role for tyrosine phosphorylation of SHP-2 in cell signaling. Mol. Cell *8*, 759–769.

Lucas, C.L., Kuehn, H.S., Zhao, F., Niemela, J.E., Deenick, E.K., Palendira, U., Avery, D.T., Moens, L., Cannons, J.L., Biancalana, M., et al. (2014). Dominant-activating germline mutations in the gene encoding the PI(3)K catalytic subunit p110δ result in T cell senescence and human immunodeficiency. Nat. Immunol. *15*, 88–97.

Luo, W., Slebos, R.J., Hill, S., Li, M., Brábek, J., Amanchy, R., Chaerkady, R., Pandey, A., Ham, A.-J.L., and Hanks, S.K. (2008). Global impact of oncogenic Src on a phosphotyrosine proteome. J. Proteome Res. *7*, 3447–3460.

Macintyre, A.N., Finlay, D., Preston, G., Sinclair, L.V., Waugh, C.M., Tamas, P., Feijoo, C., Okkenhaug, K., and Cantrell, D.A. (2011). Protein kinase B controls transcriptional programs that direct cytotoxic T cell fate but is dispensable for T cell metabolism. Immunity *34*, 224–236.

Maksumova, L., Le, H.T., Muratkhodjaev, F., Davidson, D., Veillette, A., and Pallen, C.J. (2005). Protein tyrosine phosphatase alpha regulates Fyn activity and Cbp/PAG phosphorylation in thymocyte lipid rafts. J. Immunol. *175*, 7947–7956.

Marth, J.D., Cooper, J.A., King, C.S., Ziegler, S.F., Tinker, D.A., Overell, R.W., Krebs, E.G., and Perlmutter, R.M. (1988). Neoplastic transformation induced by an activated lymphocyte-specific protein tyrosine kinase (pp56lck). Mol. Cell. Biol. *8*, 540–550.

Mitra, S., Ring, A.M., Amarnath, S., Spangler, J.B., Li, P., Ju, W., Fischer, S., Oh, J., Spolski, R., Weiskopf, K., et al. (2015). Interleukin-2 activity can be fine tuned with engineered receptor signaling clamps. Immunity *42*, 826–838.

Mizuno, K. (2013). Signaling mechanisms and functional roles of cofilin phosphorylation and dephosphorylation. Cell. Signal. *25*, 457–469.

Najafov, A., Shpiro, N., and Alessi, D.R. (2012). Akt is efficiently activated by PIF-pocket- and PtdIns(3,4,5)P3-dependent mechanisms leading to resistance to PDK1 inhibitors. Biochem. J. *448*, 285–295.

Navarro, M.N., Goebel, J., Feijoo-Carnero, C., Morrice, N., and Cantrell, D.A. (2011). Phosphoproteomic analysis reveals an intrinsic pathway for the regulation of histone deacetylase 7 that controls the function of cytotoxic T lymphocytes. Nat. Immunol. *12*, 352–361.

Navarro, M.N., Feijoo-Carnero, C., Arandilla, A.G., Trost, M., and Cantrell, D.A. (2014). Protein kinase D2 is a digital amplifier of T cell receptor-stimulated diacylglycerol signaling in naïve $CD8^+$ T cells. Sci. Signal. *7*, ra99.

Nika, K., Soldani, C., Salek, M., Paster, W., Gray, A., Etzensperger, R., Fugger, L., Polzella, P., Cerundolo, V., Dushek, O., et al. (2010). Constitutively active Lck kinase in T cells drives antigen receptor signal transduction. Immunity *32*, 766–777.

O'Shea, J.J., Schwartz, D.M., Villarino, A.V., Gadina, M., McInnes, I.B., and Laurence, A. (2015). The JAK-STAT pathway: impact on human disease and therapeutic intervention. Annu. Rev. Med. *66*, 311–328.

Okkenhaug, K., Ali, K., and Vanhaesebroeck, B. (2007). Antigen receptor signalling: a distinctive role for the p110delta isoform of PI3K. Trends Immunol. *28*, 80–87.

Osinalde, N., Moss, H., Arrizabalaga, O., Omaetxebarria, M.J., Blagoev, B., Zubiaga, A.M., Fullaondo, A., Arizmendi, J.M., and Kratchmarova, I. (2011). Interleukin-2 signaling pathway analysis by quantitative phosphoproteomics. J. Proteomics *75*, 177–191.

Osinalde, N., Sanchez-Quiles, V., Akimov, V., Guerra, B., Blagoev, B., and Kratchmarova, I. (2015). Simultaneous dissection and comparison of IL-2 and IL-15 signaling pathways by global quantitative phosphoproteomics. Proteomics *15*, 520–531.

Pipkin, M.E., Sacks, J.A., Cruz-Guilloty, F., Lichtenheld, M.G., Bevan, M.J., and Rao, A. (2010). Interleukin-2 and inflammation induce distinct transcriptional programs that promote the differentiation of effector cytolytic T cells. Immunity *32*, 79–90.

Pircher, H., Bürki, K., Lang, R., Hengartner, H., and Zinkernagel, R.M. (1989). Tolerance induction in double specific T-cell receptor transgenic mice varies with antigen. Nature *342*, 559–561.

Preston, G.C., Sinclair, L.V., Kaskar, A., Hukelmann, J.L., Navarro, M.N., Ferrero, I., MacDonald, H.R., Cowling, V.H., and Cantrell, D.A. (2015). Single cell tuning of Myc expression by antigen receptor signal strength and interleukin-2 in T lymphocytes. EMBO J. *34*, 2008–2024.

Ray, J.P., Staron, M.M., Shyer, J.A., Ho, P.-C., Marshall, H.D., Gray, S.M., Laidlaw, B.J., Araki, K., Ahmed, R., Kaech, S.M., and Craft, J. (2015). The

interleukin-2-mTORc1 kinase axis defines the signaling, differentiation, and metabolism of T helper 1 and follicular B helper T cells. Immunity *43*, 690–702.

Safari, F., Murata-Kamiya, N., Saito, Y., and Hatakeyama, M. (2011). Mammalian Pragmin regulates Src family kinases via the Glu-Pro-Ile-Tyr-Ala (EPIYA) motif that is exploited by bacterial effectors. Proc. Natl. Acad. Sci. USA *108*, 14938–14943.

Smith, G.A., Uchida, K., Weiss, A., and Taunton, J. (2016). Essential biphasic role for JAK3 catalytic activity in IL-2 receptor signaling. Nat. Chem. Biol. *12*, 373–379.

Spangler, J.B., Tomala, J., Luca, V.C., Jude, K.M., Dong, S., Ring, A.M., Votavova, P., Pepper, M., Kovar, M., and Garcia, K.C. (2015). Antibodies to interleukin-2 elicit selective T cell subset potentiation through distinct conformational mechanisms. Immunity *42*, 815–825.

Stone, E.L., Pepper, M., Katayama, C.D., Kerdiles, Y.M., Lai, C.-Y., Emslie, E., Lin, Y.C., Yang, E., Goldrath, A.W., Li, M.O., et al. (2015). ICOS coreceptor signaling inactivates the transcription factor FOXO1 to promote Tfh cell differentiation. Immunity *42*, 239–251.

Tanaka, M., Maeda, K., and Nakashima, K. (1995). Chicken alpha-enolase but not beta-enolase has a Src-dependent tyrosine-phosphorylation site: cDNA cloning and nucleotide sequence analysis. J. Biochem. *117*, 554–559.

Titz, B., Low, T., Komisopoulou, E., Chen, S.S., Rubbi, L., and Graeber, T.G. (2010). The proximal signaling network of the BCR-ABL1 oncogene shows a modular organization. Oncogene *29*, 5895–5910.

Vindis, C., Teli, T., Cerretti, D.P., Turner, C.E., and Huynh-Do, U. (2004). EphB1-mediated cell migration requires the phosphorylation of paxillin at Tyr-31/Tyr-118. J. Biol. Chem. *279*, 27965–27970.

Zhang, Z., Shen, K., Lu, W., and Cole, P.A. (2003). The role of C-terminal tyrosine phosphorylation in the regulation of SHP-1 explored via expressed protein ligation. J. Biol. Chem. *278*, 4668–4674.

Zhou, Y.J., Magnuson, K.S., Cheng, T.P., Gadina, M., Frucht, D.M., Galon, J., Candotti, F., Geahlen, R.L., Changelian, P.S., and O'Shea, J.J. (2000). Hierarchy of protein tyrosine kinases in interleukin-2 (IL-2) signaling: activation of syk depends on Jak3; however, neither Syk nor Lck is required for IL-2-mediated STAT activation. Mol. Cell. Biol. *20*, 4371–4380.

Immunity, Volume *45*

Supplemental Information

Phosphoproteomic Analyses of Interleukin 2 Signaling Reveal Integrated JAK Kinase-Dependent and -Independent Networks in $CD8^+$ T Cells

Sarah H. Ross, Christina Rollings, Karen E. Anderson, Phillip T. Hawkins, Len R. Stephens, and Doreen A. Cantrell

Figure S1

A

	p-Peptides
Expt 1	6327
Expt 2	5284
Expt 1-2 (shared)	3461 (42.3%)

	p-Peptides
Expt 1	6327
Expt 3	5450
Expt 1-3 (shared)	3482 (41.9%)

	p-Peptides
Expt 2	5284
Expt 3	5450
Expt 2-3 (shared)	4306 (66.9%)

	Expt 1	Expt 2	Expt 3
up regulated	232 (3.66%)	228 (4.31%)	281 (5.15%)
down regulated	306 (4.83%)	262 (4.95%)	201 (3.68%)

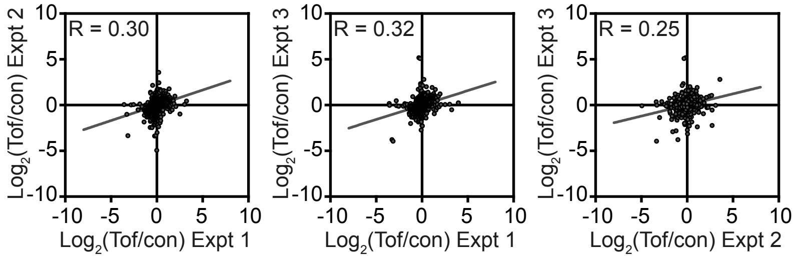

B

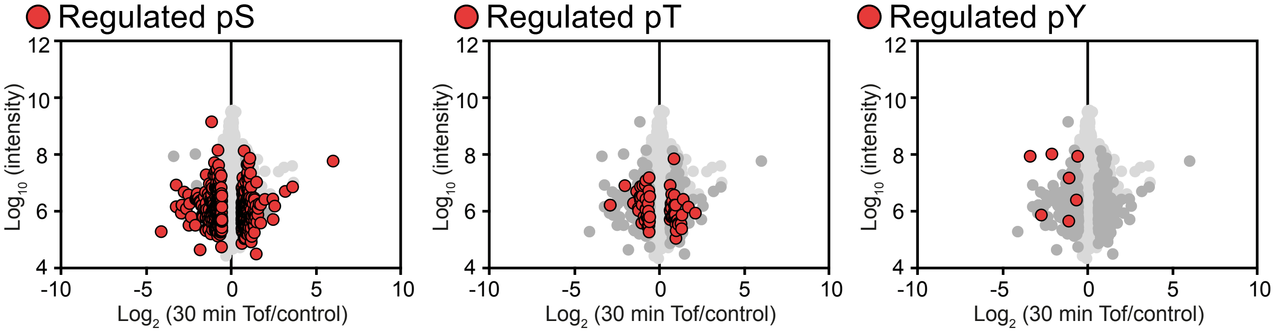

C

	p-Peptides
Expt 1	9934
Expt 2	8733
Expt 1-2 (shared)	7319 (64.5%)

	p-Peptides
Expt 1	9934
Expt 3	5941
Expt 1-3 (shared)	5101 (47.3%)

	p-Peptides
Expt 2	8733
Expt 3	5941
Expt 2-3 (shared)	5040 (52.3%)

	Expt 1	Expt 2	Expt 3
up regulated	247 (2.48%)	176 (2.01%)	98 (1.64%)
down regulated	632 (6.36%)	342 (3.91%)	258 (4.34%)

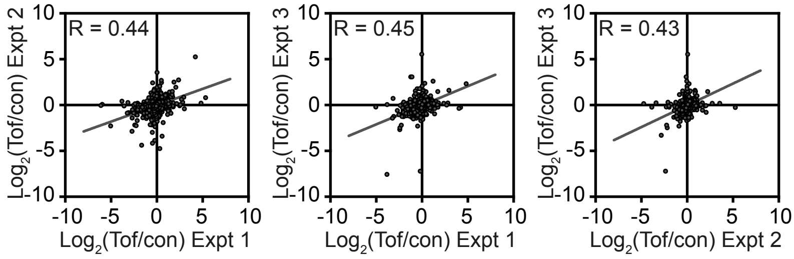

D

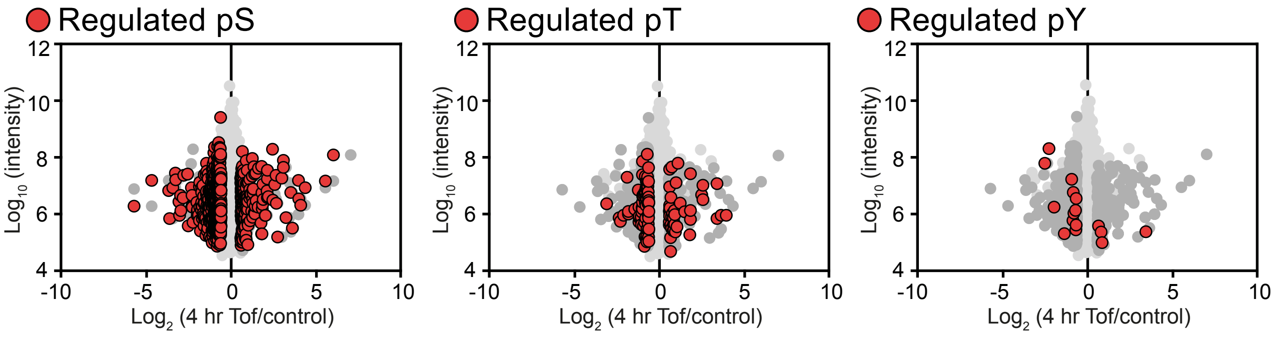

Figure S2

A

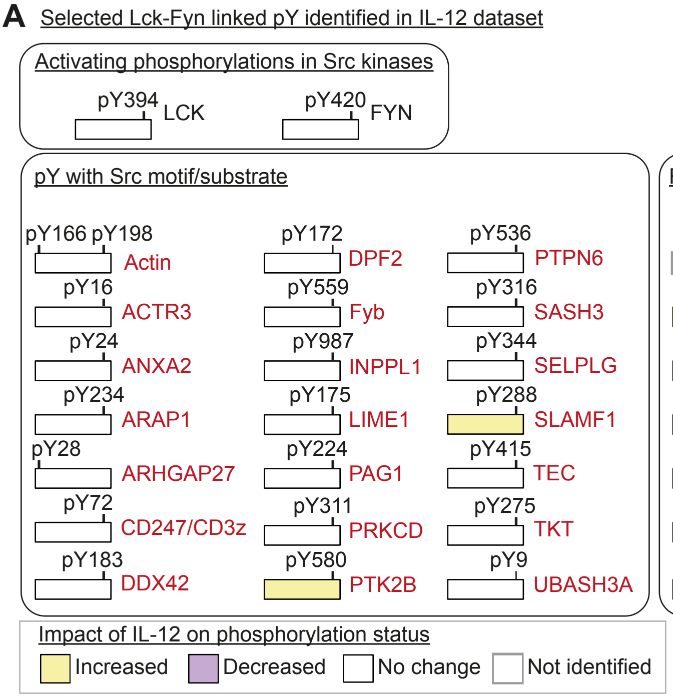

B

	p-Peptides
Expt 1	11937
Expt 2	10796
Expt 1-2 (shared)	8538 (60.1%)

	p-Peptides
Expt 1	11937
Expt 3	12183
Expt 1-3 (shared)	10271 (74.2%)

	p-Peptides
Expt 2	10796
Expt 3	12183
Expt 2-3 (shared)	8621 (60.0%)

	Expt 1	Expt 2	Expt 3
up regulated	521 (4.36%)	392 (3.63%)	615 (5.04%)
down regulated	796 (6.66%)	642 (5.94%)	878 (7.20%)

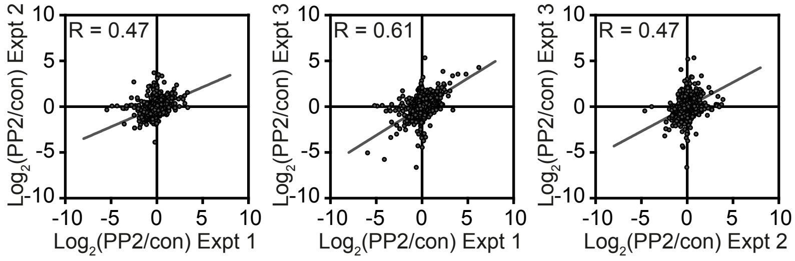

C

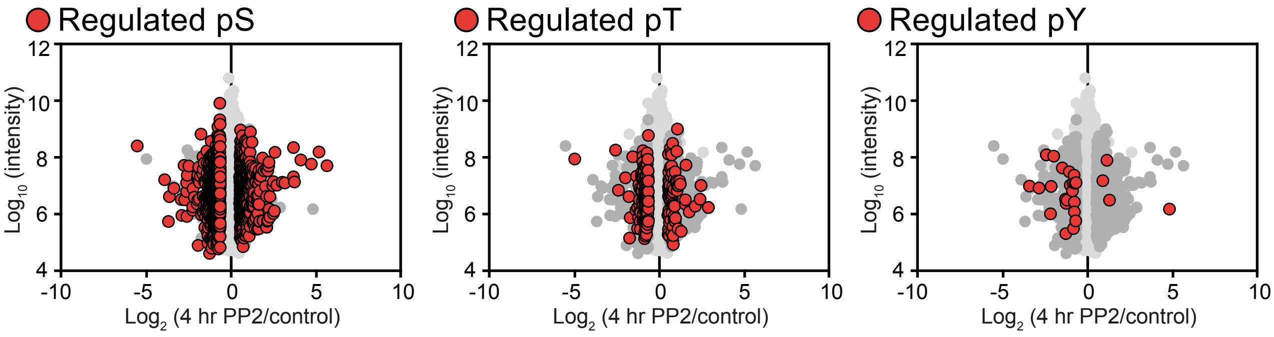

Figure S3

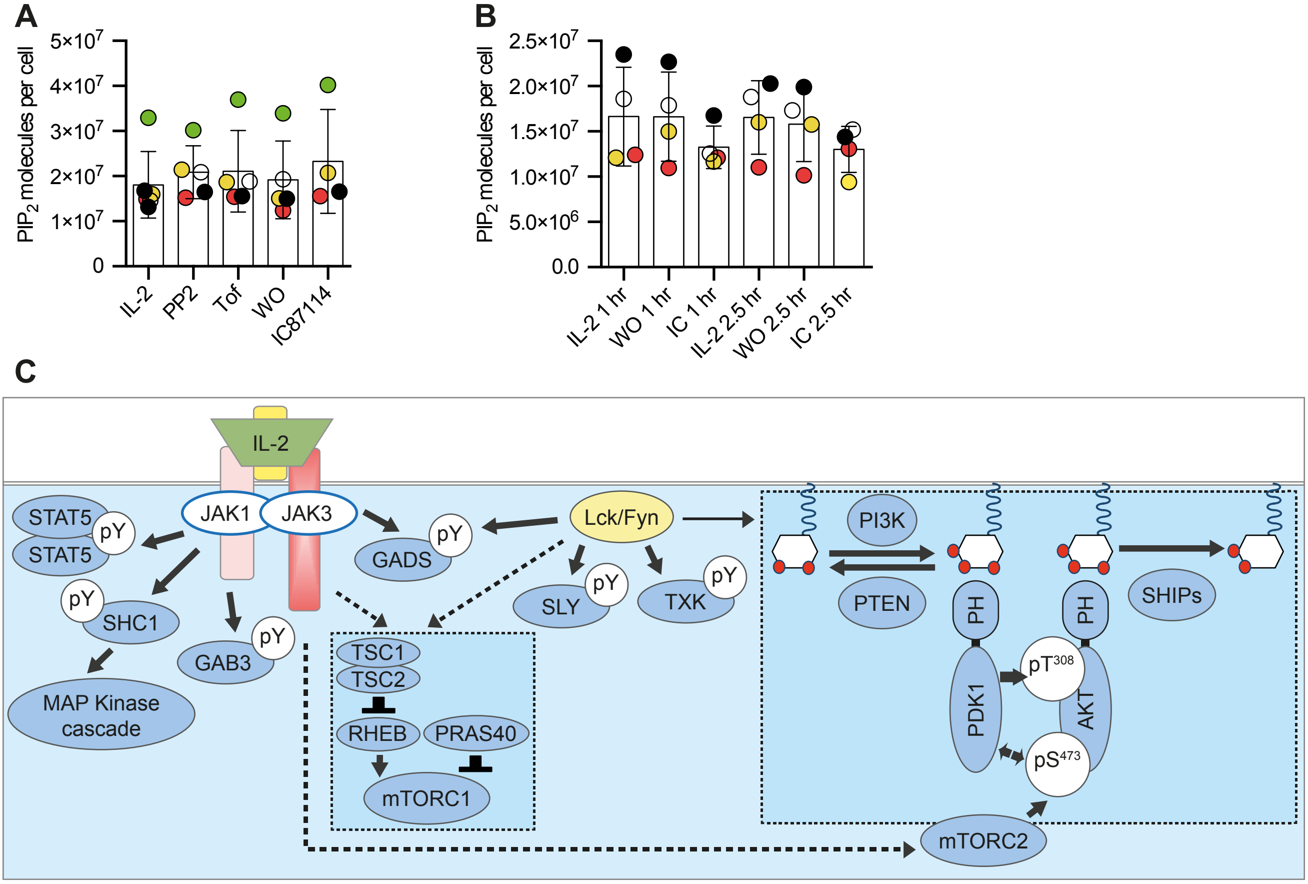
A
B
C
PIP2 molecules per cell
5×10^7
4×10^7
3×10^7
2×10^7
1×10^7
0
IL-2
PP2
Tof
WO
IC87114
2.5×10^7
2.0×10^7
1.5×10^7
1.0×10^7
5.0×10^6
0.0
IL-2 1 hr
WO 1 hr
IC 1 hr
IL-2 2.5 hr
WO 2.5 hr
IC 2.5 hr
IL-2
JAK1
JAK3
STAT5
STAT5
pY
SHC1
GAB3
MAP Kinase cascade
GADS
Lck/Fyn
SLY
TXK
TSC1
TSC2
RHEB
PRAS40
mTORC1
PI3K
PTEN
SHIPs
PH
PDK1
AKT
pT308
pS473
mTORC2

Supplemental Figure Legends

Figure S1. Related to Figure 3: Tofacitinib-regulation of the CTL phosphoproteome.
(A) The overlap and correlation in the SILAC ratios of the phosphosites identified in the individual biological replicates of IL-2 maintained CTL treated with Tofacitinib for 30 minutes. In these experiments, the "heavy"-labeled cells were treated with the inhibitor and the "light" cells were given the control treatment. The numbers and percentages of regulated phosphosites in each replicate are shown alongside. In **(B)**, a graphical representation of all the phosphosites identified in all replicates of the 30 minute Tofacitinib treatment is shown, with log-transformed SILAC ratios plotted against log-transformed peptide intensity. The individual panels show the regulated pS, pT or pY sites in red. In **(C)** the overlap and correlation in the SILAC ratios of the phosphosites identified in the individual biological replicates of IL-2 maintained CTL treated with Tofacitinib for 4 hours is shown. The numbers and percentages of regulated phosphosites in each replicate are shown alongside. The graphs in **(D)** shows the overview of all the phosphosites identified in the three biological replicates of the 4 hour Tofacitinib treatment with log-transformed SILAC ratios plotted against log-transformed peptide intensity, with the red dots in each panel showing the regulated pS, pT or pY sites.

Figure S2. Related to Figure 5: PP2-regulation of the CTL phosphoproteome.
(A) CTL differentiated in "heavy" SILAC media were treated with 20 ng/ml IL-12 for 18 hours in the presence of 20 ng/ml IL-2 and compared to "light" control, CTL maintained in 20 ng/ml IL-2. Cells were processed for mass spectrometry as described in Figure 1A and in Materials and Methods. The data were compared to the IL-2 datasets to extract the SRC kinase regulated/SRC kinase motif-containing phosphorylation sites, and the regulation of those found reproducibly in the 18 hour treatment are shown in **(A)**. **(B)** The overlap and correlation in the SILAC ratios of the phosphosites identified in the individual biological replicates of IL-2 maintained CTL treated with PP2 for 4 hours. The numbers and percentages of regulated phosphosites in each replicate are shown alongside. A graphical representation of all the phosphosites identified in the three biological replicates is shown in **(C)**, with the with log-transformed SILAC ratios plotted against log-transformed peptide intensity. The individual panels show the regulated pS, pT or pY sites in red.

Figure S3. Related to Figure 7: Control of inositol lipid levels in CTL
Mass spectrometry was used to measure the inositol lipid levels in CTL. The average number of molecules of PIP_2 per cell in response to each of the inhibitor treatments was calculated using the mass of the PIP_2 internal standard (ISD), a breakdown product of the PIP_3 ISD. The graph in **(A)** shows the number of PIP_2 per CTL in either IL-2 maintained CTL, or IL-2 maintained CTL treated with 100 nM Tofacitinib (Tof), 10 μM PP2, 10 μM IC87114 or deprived of IL-2 (WO) for 30 minutes. The results for 5 biological replicates are shown. In **(B)** levels of PIP_2 were measured in IL-2 maintained CTL, or those deprived of IL-2 (WO) or treated with IL-2 + IC87114 for up to 2.5 hours. The graph shows the data for 4 biological replicates. In **(A)** and **(B)** individual biological replicate data is shown in matching colors, the bars show the mean ± SD. **(C)** IL-2 dependent-JAK activation stimulates the phosphorylation of a number of signaling scaffold proteins and regulates diverse pT/pS signaling networks in CTL. Cell intrinsic LCK/FYN activity also regulates a number of signaling molecules, notably dominating the regulation of PIP_3 accumulation and the activity of AKT. Signaling integration between JAK and LCK/FYN occurs at multiple levels to regulate signaling that defines T cell function**.**

Supplemental Table Legends

Table S1. Related to Figure 1: The phosphoproteome of IL-2 stimulated CTL.
All the phosphosites identified in three biological replicates of CTL comparing the phosphoproteome of IL-2 quiesced CTL (con) with IL-2 quiesced CTL treated with 20 ng/ml IL-2 for 15 minutes (IL-2). Phosphosites ratios increased by 1.5-fold or greater are colored yellow and those decreased by 1.5-fold or greater are highlighted in purple. The significance column shows the log-transformed *P* values (one sample t-test). Values ≥ 1.3 are equivalent to *P* value ≤ 0.05.

Table S2. Related to Figure 1 and Figure 2: The IL-2 regulated CTL phosphoproteome.
The phosphosites reproducibly regulated in two or three biological replicates of CTL comparing the phosphoproteome of IL-2 quiesced CTL (con) with quiesced CTL treated with 20 ng/ml IL-2 for 15 minutes (IL-2) are shown. Phosphosites ratios increased by 1.5-fold or greater are colored yellow and

those decreased by 1.5-fold or greater are colored in purple. The significance column shows the log-transformed *P* values (one sample t-test). Values ≥ 1.3 are equivalent to *P* value ≤ 0.05.

Table S3. Related to Figure 1: Functional analysis of IL-2-regulated proteins.
Groupings of proteins whose phosphorylation was reproducibly regulated by IL-2 in two or three biological replicates using DAVID cluster enrichment analysis.

Table S4. Related to Figure 3: The phosphoproteome of IL-2 maintained CTL after 30 minutes of Tofacitinib treatment.
All the phosphosites identified in three biological replicates of CTL comparing the phosphoproteome of IL-2 maintained CTL (con) with IL-2 maintained CTL treated with Tofacitinib (Tof) for 30 minutes. Phosphosites ratios increased by 1.5-fold or greater are colored yellow and those decreased by 1.5-fold or greater are highlighted in purple. The significance column shows the log-transformed *P* values (one sample t-test). Values ≥ 1.3 are equivalent to *P* value ≤ 0.05.

Table S5. Related to Figure 3: The phosphoproteome of IL-2 maintained CTL after 4 hours of Tofacitinib treatment.
All the phosphosites identified in three biological replicates of CTL comparing the phosphoproteome of IL-2 maintained CTL (con) with IL-2 maintained CTL treated with Tofacitinib (Tof) for 4 hours. Phosphosites ratios increased by 1.5-fold or greater are colored yellow and those decreased by 1.5-fold or greater are highlighted in purple. The significance column shows the log-transformed *P* values (one sample t-test). Values ≥ 1.3 are equivalent to *P* value ≤ 0.05.

Table S6. Related to Figure 3: Tofacitinib-regulated phosphosites in CTL.
Lists of phosphosites identified as being regulated by Tofacitinib treatment of IL-2 maintained CTL. Phosphosites were included if they were regulated consistently in both datasets, consistently regulated in the 30 minute dataset but not detected in the 4 hour dataset, consistently regulated following the long-term Tofacitinib treatment but unchanged after 30 minutes of Tofacitinib treatment or if they were consistently regulated after the 4 hour treatment but not identified in the 30 minute treatment. Phosphosites ratios increased by 1.5-fold or greater are colored yellow and those decreased by 1.5-fold or greater are highlighted in purple. The significance columns show the log-transformed *P* values (one sample t-test). Values ≥ 1.3 are equivalent to *P* value ≤ 0.05.

Table S7. Related to Figure 1 and Figure 3: The IL-2 independent phosphoproteome.
The phosphosites identified in the IL-2 stimulated dataset or Tofacitinib datasets but that were not found to be perturbed by the treatments are shown.

Table S8. Related to Figure 4: The CTL tyrosine phosphoproteome.
All phosphotyrosine sites identified in the IL-2 stimulated dataset or Tofacitinib datasets are listed. Those with SRC family kinase motifs, or having been experimentally annotated as being SRC family substrates in the Phosphosite and ELM databases, are highlighted in yellow.

Table S9. Related to Figure 5: The phosphoproteome of IL-2 maintained CTL after 4 hours of PP2 treatment.
All the phosphosites identified in three biological replicates of CTL comparing the phosphoproteome of IL-2 maintained CTL (con) with IL-2 maintained CTL treated with PP2 for 4 hours. Phosphosites ratios increased by 1.5-fold or greater are colored yellow and those decreased by 1.5-fold or greater are highlighted in purple. The significance columns show the log-transformed *P* values (one sample t-test). Values ≥ 1.3 are equivalent to *P* value ≤ 0.05.

Table S10. Related to Figure 5: The PP2 regulated CTL phosphoproteome.
The phosphosites reproducibly regulated in two or three biological replicates of CTL comparing the phosphoproteome of IL-2 maintained CTL (con) with IL-2 maintained CTL treated with PP2 for 4 hours are listed. Phosphosites ratios increased by 1.5-fold or greater are colored yellow and those decreased by 1.5-fold or greater are colored in purple. The significance columns show the log-transformed *P* values (one sample t-test). Values ≥ 1.3 are equivalent to *P* value ≤ 0.05.

Table S11. Related to Figure 6: The integration of IL-2 and LCK/FYN mediated signaling pathways.
The phosphosites reproducibly regulated by both 4 hours of Tofacitinib (Tof) and 4 hours of PP2 treatment. Phosphosites ratios increased by 1.5-fold or greater are colored yellow and those decreased by 1.5-fold or greater are colored in purple.

Supplemental Experimental Procedures

Inhibitors and cell treatments
Tofacitinib (from Selleckchem, Munich, Germany) was used at a concentration of 100 nM. The AKT-1/2 inhibitor (AKTi) (EMD Millipore) was used at 1 μM, the PI3K-p110δ inhibitor IC87114 (made in-house) and PP2 (Tocris) were used at 10 μM. Where appropriate, cells were treated with DMSO as controls. To deprive cells of IL-2, cells were pelleted, washed in pre-warmed media lacking IL-2 for 5 minutes before being pelleted and resuspended in pre-warmed media lacking IL-2. To standardize, the time course of IL-2 deprivation started when the first media wash lacking IL-2 was added to the cells.

Cell counts
Accurate cell counts for mixing "light" and "heavy" labeled cells for the phosphoproteomic studies were obtained using a FACSVerse flow cytometer with FACSuite software (BD Biosciences). Viable cells were gated according to their forward- and side-scatter profiles and counts were determined using the FACSuite software or with FlowJo software (Treestar).

Sample preparation, phosphopeptide enrichment and mass spectrometry
For the IL-2 stimulation of CTL, cells were labeled in "light" or "heavy" media supplemented with 20 ng/ml IL-2 for 4 days. Cells were then starved of IL-2 for 24 hours. To remove IL-2, cells were washed once in pre-warmed Lysine and Arginine free media lacking IL-2, before being resuspended in the appropriated "light" or "heavy" media lacking IL-2. Cells were supplemented with 20 ng/ml IL-12 (R&D Systems) during this time to support cell viability and the expression of the IL-2 receptor alpha chain. After 24 hours, the "heavy" cells were treated with 20 ng/ml IL-2 for 15 minutes. The control, "light" samples were given a mock stimulation.

For the Tofacitinib and PP2 studies, three independent biological replicates of P14 CTLs maintained in 20 ng/ml IL-2 only were analysed. The "light" condition was used for the control cells, and the P14 CTL labeled in "heavy" SILAC media for 5 days were treated with the inhibitor.

Following the treatments, the control and treated cells were mixed at 1:1 ratio (total $2x10^8$ cells). Cells were pelleted and washed once in cold PBS and lysed in 8 M urea, 50 mM Tris-HCl pH 8 and 1 mM TCEP (Pierce). The lysed samples were then sonicated and the proteins were precipitated with trichloroacetic acid (10%, v/v) for 15 minutes at room temperature. The resulting protein pellets were washed thoroughly before being resuspended in 8M urea, 50 mM Tris-HCl pH 8. The proteins were then subjected to alkylation with iodoacetamide (Sigma-Aldrich) prior to digestion with trypsin (Promega). Proteolytic digestion products were desalted using C18 Sep-Pak cartridges (Waters), and fractionated by hydrophilic interaction liquid chromatography (HILIC).

Hydrophilic interaction liquid chromatography (HILIC) and phosphopeptide enrichment.
Desalted tryptic peptides were fractionated using Ultimate 3000 HPLC (Thermo Scientific) equipped with a 4.6 × 250- mm TSKgel Amide-80 5-μm particle column (Tosoh Biosciences). For the separation, the buffers used were 0.1% TFA (HILIC buffer A) and 99.9% acetonitrile, 0.1% TFA (HILIC buffer B). The peptide samples were resuspended in 80% HILIC buffer B and injected onto the HILIC column. The chromatography was performed using the following elution gradient: 80% B held for 20 minutes followed by 80% B to 60% B in 40 minutes and finally 0% B for 10 minutes at a flow rate of 0.4 ml/minute. In total, 16 phosphopeptide fractions were collected.

Phosphopeptides were enriched using previously described protocols. For the Tofacitinib and PP2 treatments, titanium dioxide (Titansphere, GL Science) (Larsen et al., 2005; Thingholm et al., 2006) was used for the enrichment and for IL-2 dataset Ti-IMAC (MagReSyn) (Tape et al., 2014) was used. In each case, phosphopeptides were eluted with 200 μl 0.4 M NH_4OH followed with 200 μl 0.2 M NH_4OH/50% acetonitrile and then dried using a speedvac (Genevac).

Liquid Chromatography-Mass Spectrometry
Phosphopeptide samples were resuspended in 1% formic acid and separated by nanoscale C18 reverse-phase liquid chromatography (Ultimate 3000 RSLC nano system, Thermo Scientific). The buffers used for chromatography were: HPLC Buffer A (2% acetonitrile, 0.1% formic acid), HPLC Buffer B (80% acetonitrile, 0.08% formic acid) and HPLC Buffer C (0.1% formic acid). Samples were washed onto the column with HPLC Buffer C and eluted with the following buffer gradient: 2% B (0-3 minutes), 2-40% B (3-128 minutes), 40-98% B (128-130 minutes), 98% B (130-150 minutes), 98-2% B (150-151 minutes), and equilibrated in 2% B (151-180 minutes) at a flow rate of 0.3 μl/minute. The eluting peptide solution was automatically electrosprayed into the coupled Linear Trap Quadrupole (LTQ)-Orbitrap mass spectrometer (LTQ-Orbitrap Velos Pro; Thermo Scientific) using an Easy-Spray nanoelectrospray ion source (Thermo Scientific). The mass spectrometers were operated in positive ion mode and were used in data-dependent acquisition modes. A full scan (FT-MS) was acquired at a target value of 1,000,000 ions with resolution R = 60,000 over a mass range of 335-1800 atomic mass unit (amu). The fifteen most intense ions were selected for fragmentation in the LTQ Orbitrap Velos. Fragmentation in the LTQ was induced by collision-induced dissociation (CID) with a target value of 10,000 ions. For accurate mass measurement, the "lock mass" function (lock mass = 445.120024 Da) was enabled for MS scan modes. To improve the fragmentation of phosphopeptides, the multistage activation algorithm in the Xcalibur software was enabled for each MS/MS spectrum using the neutral loss values of 97.98, 48.99, 32.66 and 24.49 m/z units. Former target ions selected for MS/MS were dynamically excluded for 45 seconds. General mass spectrometric conditions were as follows: spray voltage, 1.8-2.5 kV; no sheath and auxiliary gas flow; ion transfer tube temperature, 250 °C; normalised collision energy (35%) using wide band activation mode for MS2. The isolation width was set to 2 amu for IT-MS/MS. Ion selection thresholds were 5000 counts for MS2. An activation of q = 0.25 and activation time of 10 ms were applied in MS2 acquisitions. The fill time for FTMS was set to 500 ms and for ITMS to 100 ms.

Data processing and Bioinformatics
Mass spectrometry data were processed in MaxQuant version 1.5.0 using the following search parameters: a MS tolerance of 20 ppm, MS/MS tolerance of 0.5 Da and full trypsin specificity. Up to two missed cleavages were permitted. Protein N-terminal acetylation, oxidation of methionine, glutamine to pyroglutamate conversion, deamidation (NQ) and phosphorylation of serine, threonine and tyrosine were set as variable modifications, while carbamidomethylation of cysteine was set as fixed modification. The minimum peptide length for identification was set to at least 6 amino acids in length. The match between run function was enabled. False discovery rates (FDRs) of 0.01 were based on hits against a reversed sequence database and calculated at the level of peptides, proteins and modification sites.

Perseus software was used to map identified phosphorylation sites with kinase motifs, and known kinase-substrate interactions obtained from the mouse and human PhosphoSitePlus (Hornbeck et al., 2015) database and the human phosphoELM (Dinkel et al., 2016) database. In addition, Perseus software was used to annotate the identified phosphoproteins with Gene Ontology (GO) biological processes (BP) and molecular functions (MF).

Phosphosites were considered to be up regulated if the SILAC ratio was greater than 1.5 and down regulated if the SILAC ratio was less than 0.667. Reproducibly regulated phosphorylation sites had to be identified in at least two of the three biological replicates and regulated consistently where identified. Statistical analysis of the Log_2 transformed ratio changes was performed using a one-sample t-test in Perseus. Regulated phosphorylation sites were not selected based on a statistical cut-off to ensure that all potentially regulated sites would be considered when comparing datasets. When phosphosites were identified in multiple biological replicates, the mean SILAC ratio of the replicates was used to present data.

Phosphosites considered to be regulated by Tofacitinib were either regulated consistently in both the 30 minute and 4 hour treatment datasets; consistently regulated in the 30 minute dataset but not detected in the 4 hour dataset; consistently regulated following the long-term Tofacitinib treatment but unchanged after 30 minutes of Tofacitinib treatment; consistently regulated after the 4 hour treatment but not identified in the 30 minute treatment.

When comparing the impact of different treatments on the CTL phosphoproteome, only phosphorylation sites reproducibly identified in both datasets were compared.

MaxQuant reports phosphosite positions for all potential isoforms of the relevant protein: in figures, where possible, the numbering for the first protein isoform is shown rather than the first phosphosite reported in the data table.

The proteins identified as being regulated by each treatment were subjected to functional analysis using DAVID bioinformatics resources to search for associated GO-BP and GO-MF terms. For enrichment analysis using DAVID, the proteins regulated by each treatment were compared to a custom background comprised of all phosphoproteins identified in the dataset (regulated and non-regulated). The combined BP and MF GO annotations were then grouped using the functional annotation clustering tool, with a medium clustering stringency and an EASE (Expression analysis systematic explorer) score, a modified Fisher Exact *P*-value, of ≤0.1.

Western blotting
Cells were lysed in ice cold lysis buffer (50 mM HEPES, pH 7.4, 150 mM sodium chloride, 1% (w/v) NP-40, 0.5% (w/v) sodium deoxycholate, 0.1% (w/v) SDS, 10% (w/v) glycerol, 1 mM EDTA, 50 mM sodium fluoride, 5 mM sodium pyrophosphate, 10 mM sodium β-glycerophosphate, 0.5 mM sodium orthovanadate, 5 mM N-ethylmaleimide, 1 mM TCEP (Pierce), with protease and phosphatase inhibitor tablets (Roche) at a concentration of 20 million CTL per ml lysis buffer. Lysates were sonicated on ice and centrifuged (16,000.g for 10 minutes at 4°C) before being mixed with NuPAGE LDS sample buffer (Life Technologies) supplemented with TCEP and separated by gel electrophoresis (Bio-Rad Mini-PROTEAN tetra cell system). Proteins were then transferred to nitrocellulose membrane (Protran), using standard conditions (Hukelmann et al., 2016). Membranes were blocked with 5% (w/v) Bovine Serum albumen and 1% (w/v) non-fat dried skimmed milk powder in phosphate-buffered saline (PBS) containing 0.05% Tween 20.

Membranes were probed with the following primary antibodies: STAT5A/B (CST #9363), STAT5A/B pY694/699 (CST #9351), S6 pS235/236 (CST #2211), YB-1 (YBX1) pS102 (CST #2900), AKT (CST #9272), AKT pT308 (CST #4056), AKT pS473 (CST #4058), SMC1 (Bethyl Laboratories, A300-055A), FOXO1/3A pT24/32 (CST #9464), FOXO1 (CST #9454), p44/42 (Erk1/2) (clone 3A7, CST #9107), p44/42 (ERK1/2) pT202/pY204 (clone E10, CST #9106). Primary antibodies were detected using HRP-conjugated secondary antibodies (goat anti rabbit, Thermo Scientific #31460; goat anti mouse, Thermo Scientific #31430), and chemiluminescence was measured using X-ray films (Konica) or an Odyssey Fc Imaging System (Licor).

Cell staining and flow cytometry
For surface markers, cells were washed once in 0.5% FBS (v/v) in PBS and stained for 20 minutes at 4°C in the same solution with saturating concentrations of antibodies as described previously (Navarro et al., 2012). Antibodies used were CD8α (clone 53-6.7) coupled to Horizon V450 (HV450) and CD25 (clone PC61) coupled to phycoerythrin-cyanine 7 (PE-Cy7) from BD Biosciences.

For intracellular staining of phospho-S6, cells were treated with or without inhibitors as appropriate, with control cells being treated 20 nM rapamycin for 30 minutes. Following treatments, cells were fixed in 1% (w/v) paraformaldehyde at 37ºC for 15 minutes. Cells were then washed in 0.5% FBS (v/v) in PBS removed from the fixation buffer and permeabilised and incubated with 90% (v/v) methanol at -20ºC for at least 30 minutes. Following permeabilisation, cells were washed twice and incubated with antibody against S6 pS235/pS236 (CST #2211) for 30 minutes at room temperature. Following staining, cells were washed twice and incubated with Alexa 647-conjugated anti-rabbit secondary antibody (CST #4414) for 30 minutes at room temperature. Cells were washed twice and resuspended in 0.5% FBS (v/v) in PBS for acquisition.

For O-propargyl-puromycin (OPP, Jena Bioscience) incorporation analysis, cells were stimulated as appropriate, with control cells being pre-treated with cycloheximide for 30 minutes. Following labeling with 20 μM OPP for 10 minutes, cells were fixed with 1% paraformaldehyde for 15 minutes at room temperature. Cells were washed in 0.5% FBS (v/v) in PBS before being permeabilised with 0.5% Triton X-100 (v/v) in PBS for 15 minutes at room temperature. The incorporated OPP was then labeled with Alexa 647-azide (Invitrogen) using a standard Click-IT chemistry reaction (Invitrogen). Cells were then washed twice with 0.5% FBS (v/v) in PBS, before being analysed.

Data were acquired on a FACS LSR Fortessa flow cytometer with DIVA software (BD Biosciences) or FACSVerse flow cytometer with FACSuite software (BD Biosciences). Viable cells were gated according to their forward- and side-scatter profiles. Data analysis was performed with FlowJo software (Treestar).

Mass spectrometry measurements of inositol lipids
The analysis of inositol lipids was performed as described previously (Clark et al., 2011), using a QTRAP 4000 (AB Sciex) mass spectrometer and employing the lipid extraction and derivatization method described for cultured cells, with the modification that 10 ng C17:0/C16:0 PIP_3 internal standard (ISD) and 100 ng C17:0/C16:0 phosphatidylinositol (PI) ISD were added to primary extracts, and that final samples were dried in a speedvac concentrator rather than under N_2. PIP_3 responses were normalised to PIP_2 responses. The mass of the PIP_2 ISD, a breakdown product of the PIP_3 ISD, was calculated using the PIP_3 internal standard (ISD), and then was used to calculated the average number of PIP_2 molecules per cell in response to each of the inhibitor treatments.

Confocal microscopy
Cells were allowed to attach to coverslips coated with poly-l-lysine (Sigma-Aldrich), then fixed for 30 minutes at 25°C with 4% (w/v) paraformaldehyde. Following fixation, cells were permeabilised using 0.1% Triton X-100 for 5 minutes. Actin was visualized using phalloidin coupled to Alexa fluor 647 (Invitrogen). A Zeiss LSM700 confocal microscope with an alpha Plan-FLUAR 100× objective (numerical aperture, 1.45) was used for imaging.

Supplemental References

Clark, J., Anderson, K.E., Juvin, V., Smith, T.S., Karpe, F., Wakelam, M.J.O., Stephens, L.R., and Hawkins, P.T. (2011). Quantification of PtdInsP3 molecular species in cells and tissues by mass spectrometry. Nat. Methods *8*, 267–272.

Dinkel, H., Van Roey, K., Michael, S., Kumar, M., Uyar, B., Altenberg, B., Milchevskaya, V., Schneider, M., Kühn, H., Behrendt, A., et al. (2016). ELM 2016--data update and new functionality of the eukaryotic linear motif resource. Nucleic Acids Res. *44*, D294–D300.

Hornbeck, P.V., Zhang, B., Murray, B., Kornhauser, J.M., Latham, V., and Skrzypek, E. (2015). PhosphoSitePlus, 2014: mutations, PTMs and recalibrations. Nucleic Acids Res. *43*, D512–D520.

Hukelmann, J.L., Anderson, K.E., Sinclair, L.V., Grzes, K.M., Murillo, A.B., Hawkins, P.T., Stephens, L.R., Lamond, A.I., and Cantrell, D.A. (2016). The cytotoxic T cell proteome and its shaping by the kinase mTOR. Nat. Immunol. *17*, 104–112.

Larsen, M.R., Thingholm, T.E., Jensen, O.N., Roepstorff, P., and Jørgensen, T.J.D. (2005). Highly selective enrichment of phosphorylated peptides from peptide mixtures using titanium dioxide microcolumns. Mol. Cell Proteomics *4*, 873–886.

Navarro, M.N., Sinclair, L.V., Feijoo-Carnero, C., Clarke, R., Matthews, S.A., and Cantrell, D.A. (2012). Protein kinase D2 has a restricted but critical role in T-cell antigen receptor signalling in mature T-cells. Biochem. J. *442*, 649–659.

Tape, C.J., Worboys, J.D., Sinclair, J., Gourlay, R., Vogt, J., McMahon, K.M., Trost, M., Lauffenburger, D.A., Lamont, D.J., and Jørgensen, C. (2014). Reproducible automated phosphopeptide enrichment using magnetic TiO2 and Ti-IMAC. Anal Chem *86*, 10296–10302.

Thingholm, T.E., Jørgensen, T.J.D., Jensen, O.N., and Larsen, M.R. (2006). Highly selective enrichment of phosphorylated peptides using titanium dioxide. Nat Protoc *1*, 1929–1935.
